# Heterophyllin B enhances transcription factor EB-mediated autophagy and alleviates pyroptosis and oxidative stress after spinal cord injury

**DOI:** 10.7150/ijbs.97669

**Published:** 2024-10-07

**Authors:** Haojie Zhang, Wei Wang, Xinli Hu, Zheng Wang, Junsheng Lou, Peng Cui, Xuan Zhao, Yu Wang, Xiaolong Chen, Shibao Lu

**Affiliations:** 1 Department of Orthopedics, Xuanwu Hospital, Capital Medical University, Beijing 100053, China.; 2 National Clinical Research Center for Geriatric Diseases, Xuanwu Hospital, Capital Medical University, Beijing 100053, China.; 3 Department of Orthopedic Surgery, The First Affiliated Hospital, Zhejiang University School of Medicine, No. 79 Qingchun Road, Hangzhou 310003, China.

**Keywords:** Heterophyllin B, spinal cord injury, oxidative stress, pyroptosis, autophagy, TFEB

## Abstract

Traumatic spinal cord injury (SCI) has devastating physical, psychosocial, and vocational implications for patients and caregivers. Heterophyllin B (HB) is a brain-permeable cyclopeptide from *Pseudostellaria heterophylla* that promotes axonal regeneration and neuroinflammation. However, the efficacy of HB in improving functional recovery following SCI and the underlying mechanisms remain unclear. This study utilized a murine model for SCI assessment to evaluate the therapeutic effects of HB. following HB intervention, functional recovery post-SCI, was assessed through the Basso Mouse Scale, gait analysis, and the detection of motor-evoked potentials (MEPs). RNA sequencing was used to study the roles of pyroptosis, oxidative stress, and autophagy in HB's impact on SCI. Techniques such as Western blot, immunofluorescence, and enzyme-linked immunosorbent assay were used to evaluate pyroptosis, oxidative stress, and autophagy markers. Associated virus vectors were used to suppress transcription factor EB (TFEB), an autophagy regulator, in a living organism. HB promoted autophagy by enhancing TFEB nuclear translocation. In contrast, it inhibited pyroptosis and oxidative stress. Based on using the adenosine monophosphate-activated protein kinase (AMPK) inhibitor Compound C, the AMPK-TRPML1-calcineurin pathway was involved in HB's regulation of TFEB. In summary, this study demonstrated that HB facilitated functional recuperation by stimulating TFEB-driven autophagy while simultaneously suppressing pyroptosis and oxidative stress after SCI, indicating its potential for clinical application.

## Introduction

Traumatic injuries to the spinal cord, recognized as SCI, manifest as devastating conditions that strip individuals of their sensory, motor, and autonomic capabilities, leading to profound disability 1. The World Health Organization (WHO) provides data indicating an annual incidence rate of SCI ranging from 250,000 to 500,000 cases worldwide, underscoring the global burden of this condition 2, 3. Despite advances in understanding the pathophysiology of SCI, neuroprotective and regenerative therapeutic approaches have limited clinical applications 4. SCI manifests in two distinct stages: an immediate phase and a secondary sequence of events. Initially, the spinal cord sustains direct damage due to trauma, such as fractures and dislocations, leading to the disruption of axons and cellular membranes 5. Following this initial trauma, a complex secondary phase unfolds, characterized by a series of physiological disturbances, including localized bleeding, ischemia, hypoxia, imbalances in ion concentration, and the accumulation of oxidative stress. These disturbances contribute to a breakdown in neural regulation, initiate the process of cell death, and lead to extensive tissue damage 5, 6. While primary injury is inevitable and irreversible, secondary injury provides a rationale and therapeutic window for interventions that may interrupt delayed cell and tissue loss 7. Emphasis has been placed on reducing neuroinflammation and pro-inflammatory cell death in injuries, the key aspects of SCI intervention 8, 9.

In the aftermath of SCI, neuroinflammation acts as a precursor to a series of secondary events that dramatically reduce the chances of neuronal survival, hinder the regeneration of axons, and compromise the functional recovery process 10. Pyroptosis, an inflammatory-driven form of cell death, is orchestrated by inflammasomes and specifically executed by GSDMD, leading to the emission of pro-inflammatory agents like IL-1β and IL-18 11. Inflammasomes are typically composed of components such as NLRP3, apoptosis-associated speck-like protein with a caspase recruitment domain (ASC), and caspase-1, which collectively assemble in the presence of internal signals indicating cellular danger 12. The overproduction of reactive oxygen species (ROS), the efflux of potassium, and the escape of cathepsin are currently recognized as the principal stimuli for the activation of the NLRP3 inflammasome 13. Emerging research points to SCI as a trigger for the activation of the NLRP3 inflammasome within the microglia and neurons of the spinal cord, marking a critical pathway for secondary damage 10, 14. Targeting the NLRP3 inflammasome and its associated components thus represents a significant avenue for neuroprotection in models of SCI in mice, offering potential therapeutic strategies for mitigating the effects of SCI.

The escalation of ROS production signifies a critical aspect of secondary damage following SCI 15. The generation of ROS (such as O_2_·^-^ and ·OH^-^) and RNS (for instance, ·NO^-^) surges under oxidative stress conditions throughout the organism 16. Accompanying SCI is an overproduction of free radicals, where the oxidative burden surpasses the body's natural antioxidant defenses, triggering an oxidative stress response 16. This imbalance renders the spinal cord vulnerable to oxidative harm. Many experimental and clinical investigations have underscored the pivotal role of ROS and lipid peroxidation in the development of SCI 17, 18. Strategies tomitigate oxidative stress or minimize its destructive effects by targeting the underlying oxidative mechanisms offer promising avenues for SCI therapy.

Autophagy, a lysosomal degradation pathway for cytoplasmic components and organelles, is crucial for maintaining cellular equilibrium and defending against external stressors 19. This process has been pinpointed as a potential therapeutic target in various pathologies, including SCI 20, 21. By activating autophagy, the integrity of the blood-brain barrier can be preserved post-trauma, thereby mitigating blood cell invasion, the inflammatory cascade, and the death of neuronal cells, which in turn facilitates the restoration of neural functions 22. Moreover, studies have demonstrated that autophagy can suppress the systemic inflammatory reaction and alleviate inflammation-induced tissue damage. This is achieved through the modulation of the NOD-, LRR-, and pyrin domain-containing protein 3 inflammasome activity and the removal of mitochondrial ROS 23. Simultaneously, the significance of Transcription factor EB (TFEB), a pivotal regulator within the autophagy pathway and a member of the MiT/TFE family, has been extensively explored. TFEB orchestrates the autophagy/lysosomal-to-nucleus signaling, positioning it as a key player in this process 24. Consequently, strategies that enhance autophagic activity through the stimulation of TFEB offer promising avenues for promoting recovery from SCI.

*Pseudostellaria heterophylla*, utilized in traditional Chinese medicine, is renowned for its diverse health benefits, including enhancing endurance, alleviating stress, strengthening the immune system, acting as an antitussive, and safeguarding intestinal health 25, 26. Heterophyllin B (HB), a cyclic peptide extracted from *Pseudostellaria heterophylla*, has captured scientific interest due to its dual roles in inhibiting tyrosinase activity and diminishing inflammatory responses 27. HB significantly alleviates the impairment of splenic T helper cells induced by Aβ1-42, reduces neuroinflammation, and improves cognitive and spatial memory in AD mouse models 28. Furthermore, HB effectively counters inflammatory damage and oxidative stress induced by LPS through the inhibition of the PI3K/Akt signaling pathways 29. In addition, HB promotes the stability of intestinal epithelial cells and lessens colonic inflammation by stimulating the AMPK pathway 30. Whether HB can mitigate neuroinflammation and enhance neurological recovery following SCI remains unclear.

In our study, a mouse model was established to simulate contusion SCI, enabling the assessment of HB therapeutic efficacy. It aimed to (1) explore the capability of HB to provide neuroprotection in SCI conditions, (2) examine whether HB's mechanism for alleviating SCI involves modulating processes such as pyroptosis, oxidative stress, and autophagy mediated by TFEB, and (3) investigate the possibility of TFEB's activity being modulated through a distinct signaling pathway.

## Materials and methods

### Animals and ethics statement

This investigation utilized adult C57BL/6J mice, inclusive of both sexes, within the age range of 6-8 weeks and a weight bracket of 18-22 grams. The Animal Experimentation Ethics Committee of Xuanwu Hospital, affiliated with Capital Medical University, provided ethical approval for this study (Approval ID: 2021-0008). These mice were kept under strict environmental controls, ensuring a stable temperature of 22 ± 2 °C, adherence to a 12-hour light-dark regime, and maintaining humidity at 55 ± 10 %. Continuous access to both food and water was guaranteed.

### Contusion SCI model

Before undergoing surgical procedures, animals were first sedated with an intraperitoneal administration of 1% pentobarbital sodium, amounting to a dosage of 50 mg/kg, ensuring effective anesthesia. Following sedation, a targeted laminectomy was precisely executed at the spinal segments T9-T10, revealing the dorsal side of the spinal cord while meticulously avoiding any harm to the dura mater. Subsequently, the exposed spinal region was subjected to a contusion injury with a 70-kilodyne force exerted by an Infinite Horizons Impactor, a device provided by Precision Systems & Instrumentation (Lexington, KY, USA). After inducing the contusion, the surgical incisions were carefully closed layer by layer using sutures made of 4-0 silk. For animals allocated to the control group, identified as the Sham group, the procedure was limited to the laminectomy at the same vertebral level, without inducing any contusion. In the postoperative phase, animals were kept in an environment with controlled temperature until their ability to regulate body temperature autonomously was fully restored to ensure optimal recovery. In the days following SCI, manual assistance was provided to facilitate bladder emptying three times a day until the animals regained normal urinary function. An antibiotic regimen with gentamicin sulfate, administered intraperitoneally at a dose of 0.03 g/kg, was initiated and continued for three days post-surgery to mitigate the risk of infections.

### Treatment and groups

For the RNA sequencing analysis, the number of biological replicates was set at five (n = 5). For all other experimental assays, each group consisted of six replicates (n = 6). A total of 274 C57BL/6J mice were randomly assigned to one of nine experimental conditions: Sham (n = 30), SCI (n = 41), SCI + HB (20 mg/kg) (n = 41), SCI + 3MA (n = 24), SCI + HB/3MA (n = 24), SCI + HB/scrambled shRNA (n = 24), SCI + HB/TFEB shRNA (n = 24), SCI + Compound C (CC) (n = 12), SCI + HB/CC (n = 12), SCI + MHY1485 (n = 6), SCI + HB/MHY1485 (n = 6), SCI + tacrolimus (n = 6), SCI + HB/tacrolimus (n = 6), SCI + HB (5 mg/kg) (n = 6), SCI + HB (10 mg/kg) (n = 6), and SCI + HB (40 mg/kg) (n = 6). The procedure for the Sham group involved performing a laminectomy without inducing SCI. The mice in the SCI + HB group were treated with an intraperitoneal injection with HB dissolved in corn oil at a dosage of 20 mg/kg daily for three days post-SCI. In a similar manner, mice in the SCI + 3MA and SCI + HB/3MA conditions received a 15 mg/kg dose of 3MA in saline solution via intraperitoneal injection for three consecutive days following SCI. The SCI + CC and SCI + HB/CC groups received CC in saline solution (1.5 mg/kg) through intraperitoneal injection for three consecutive days post-SCI. For the SCI + HB/TFEB shRNA group, a standard laminectomy was performed two weeks prior to surgery at the T9-T10 level, following an intraperitoneal injection of 1% pentobarbital sodium. Micro-syringes were inserted at a 45° angle to inject viral vectors in PBS into eight designated sites within the spinal cord at a flow rate of 0.2 μL/min, with each site receiving 2 μL. The SCI + HB/scrambled shRNA group received an equivalent adeno-associated virus (AAV) vector volume. Post-injection, the mice showed no signs of hind limb paralysis or paresis. On days 3, 14, and 28 post-SCI, mice were euthanized with an overdose of pentobarbital sodium to collect tissue for histological analysis.

### Reagents

Med Chem Express (Monmouth Junction, NJ, USA) supplied HB (C_40_H_58_N_8_O_8_, 99.88% pure, catalog # HY-N1476), 3MA (C_6_H_7_N_5_, 99.91% pure, catalog # HY-19312), compound C (C_24_H_25_N_5_O, 99.91% pure, catalog # HY-13418A), MHY1485 (C_17_H_21_N_7_O_4_; 99.86% pure, cat # HY-B0795), tacrolimus (C_44_H_69_NO_12_; 99.93% pure, cat # HY-13756) and gentamicin sulfate (C_24_H_55_N_7_O_11_S_3_; catalog # HY-A0276). Reagents for hematoxylin and eosin (H&E) staining (catalog # G1120), Masson's trichrome staining (catalog # G1340), and Nissl staining (catalog # G1430) were acquired from Solarbio Science & Technology (Beijing, China). Epizyme Biomedical Technology (Shanghai, PRC) was the source for Omni-Easy™ Instant BCA Protein Assay Kits (catalog # ZJ102), Omni-Easy™ Protein Sample Loading Buffer (catalog # LT101S), and Omni-ECL™ Femto Light Chemiluminescence Kits (catalog # SQ201). Thermo Fisher Scientific (Rockford, IL, USA) provided NE-PER™ nuclear and cytoplasm extraction reagents (catalog # 78835). Abcam (Cambridge, UK) supplied the aqueous mounting medium, which includes 4'-6-diamidino-2-phenylindole (DAPI) and Fluoroshield (catalog # ab104139).

### Evaluation of the locomotive function

Motor functionality in the hind limbs was systematically evaluated at predetermined intervals employing the Basso Mouse Scale (BMS), with scores ranging from 0, indicative of complete paralysis, to 9, signifying unimpaired motor function. This evaluation focused on analyzing both the movement and coordination in the hind limbs' joints 31. The comparative analysis of locomotor capabilities among the various experimental groups was facilitated through footprint tracking, where forelimbs were marked in blue and hind limbs in red, to provide clear differentiation. The integrity of motor functions within the lower limbs' nervous system was quantitatively assessed on the 28^th^ and 56^th^ day following SCI using motor evoked potentials (MEPs), leveraging the BL-420A/F Data Acquisition and Analysis System provided by TECHMAN SOFT. This involved placing stimulating electrodes directly onto the motor cortex and strategically positioning recording electrodes on the sciatic nerve of the opposite side to capture the neurological responses. The amplitude of the initial evoked potential was selected as the key metric for assessing motor neuron performance. This assessment was conducted by a pair of researchers who were methodically blinded to the experimental conditions assigned to each mouse to ensure objectivity in the evaluation process.

### AAV vector packaging

GeneChem Chemical Technologies in Shanghai, China, supplied AAV vectors encoding TFEB shRNA (serotype 9; sequence: *CCAAGAAGGATCTGGACTT*, lacking a fluorescent reporter gene) and a control AAV vector with a scrambled shRNA sequence (serotype 9; sequence: *CGCTGAGTACTTCGAAATGTC*). The concentrations of these AAV vectors, AAV-TFEB shRNA and AAV-scrambled shRNA, were quantified through quantitative qPCR, revealing titers of 1.68 × 10¹² and 1 × 10¹² genome copies per mL, respectively.

### Western blotting

Three days post-SCI, mice were euthanized using an excess of sodium pentobarbital. Spinal cord segments measuring 5 mm in length were collected around the injury epicenter for the SCI groups, and from a corresponding region for the control group, and were immediately frozen at -80 °C. These samples underwent lysis in RIPA buffer (Beyotime) enhanced with both a protease and phosphatase inhibitor cocktail (Sigma-Aldrich). A multisample homogenizer (OMNI Prep) facilitated the homogenization of each sample for one minute per pulse, interspersed with five-minute intervals, for three to five rounds. Protein concentrations were ascertained using the Pierce BCA Protein Assay Kit (Thermo Fisher Scientific). The separation of cytoplasmic and nuclear proteins was achieved using the NE-PER kit as per established protocols. Proteins (60 μg per sample) were then electrophoresed on 12% SDS-PAGE gels and transferred onto PVDF membranes (Millipore). Membranes were pre-treated with 5% non-fat milk at ambient temperature for two hours to block non-specific binding sites, followed by an overnight exposure at 4 °C to primary antibodies directed against a spectrum of proteins such as VPS34 (1:1,000), SQSTM1/p62 (1:1,000), Beclin1 (1:1,000), CTSD (1:1,000), LC3B (1:1,000), NLRP3 (1:1,000), ASC (1:1,000), caspase-1 (1:1,000), GSDMD (1:1,000), IL-1β (1:1,000), IL-18 (1:1,000), HO1 (1:1,000), SOD1 (1:2,000), AMPK (1:1,000), phosphorylated AMPK (1:1,000), mTOR (1:2,000), phosphorylated mTOR (1:2,000), TRPML1 (1:1,000), calcineurin (1:1,000), TFEB (1:1,000), Histone-H3 (1:5,000), GAPDH (1:4,000), and β-actin (1:10,000), each at specific dilutions. The procedure proceeded with a two-hour incubation with HRP-tagged secondary antibodies, and band detection and examination were performed using Omni-ECL™ Femto Light Chemiluminescence Kits alongside the ChemiDoc™ XRS+ Imaging System provided by Bio-Rad.

### Superoxide dismutases and malondialdehyde detection

To assess oxidative stress, the level of malondialdehyde (MDA) was measured employing a TBA assay, adhering strictly to the protocol provided by Boyun Biotech in Shanghai, China. Cell samples underwent protein extraction, and a standard MDA solution was prepared. These were then mixed with the MDA-specific working solution and incubated at 100 °C for 15 minutes. This step was followed by centrifugation at 1000 × g for 10 minutes. The quantification of MDA was conducted by measuring the optical densities at 535 nm using a microplate reader. Similarly, SOD content was evaluated through a WST-8-based colorimetric assay. SODs were evaluated through a WST-8-based colorimetric assay at 450 nm with a microplate reader, as per the guidelines of Boyun Biotech.

### Dihydroethidium staining

The process involved dehydrating the spinal cord tissue in a 30% sucrose solution until it was fully submerged and sank to the bottom. Once dehydrated, the tissue was carefully laid on sterile filter paper to effectively remove any remaining moisture. It was then encased in an OCT compound, followed by cooling to ensure the OCT fully solidified, preparing the tissue for sectioning. The fully solidified block was then precision-cut into frozen sections with a thickness of 20 micrometers. To reduce autofluorescence, these sections underwent treatment with an autofluorescence quenching solution for a duration of five minutes. For the specific detection of oxidative stress, a concentrated stock solution of DHE supplied by Thermo Fisher Scientific was accurately diluted in phosphate-buffered saline to achieve the required staining concentration. This preparation was swiftly applied to the sections, which were then incubated away from light at 37 °C for half an hour. Following the incubation, the sections were washed in phosphate-buffered saline three times, each for five minutes, to remove any unbound stain, at room temperature. The examination under a fluorescence microscope highlighted the areas of interest, and the red fluorescence emitted was quantitatively measured using ImageJ software, providing a clear and objective assessment of oxidative stress markers within the tissue samples.

### RNA sequencing and analysis of differentially expressed genes

Total RNA was extracted employing TRIzol reagent (provided by Life Technologies Corp.) and underwent DNase treatment to eliminate any genomic DNA contamination. The mRNA was then purified using the NEBNext PolyA mRNA Magnetic Isolation Module (courtesy of New England Biolabs, Ipswich, MA, USA), which was subsequently utilized for constructing RNA-Seq libraries with the assistance of the NEB Next Ultra Directional RNA Library Prep Kit for Illumina (also from New England Biolabs, Ipswich, MA, USA). These libraries were analyzed using Illumina sequencing technology in a paired-end 2 × 150 sequencing format. The initial sequencing output underwent processing to generate high-quality clean reads by excising sequencing adapters, discarding reads shorter than 35 bp, and filtering out low-quality sequences with the aid of Cutadapt and Trimmomatic. Quality control checks on the refined reads were conducted using FastQC. These optimized reads were aligned to the mouse genome reference (GRCm38.p6) using HISAT2. Estimations of gene expression levels were calculated as FPKM employing StringTie. Differential gene expression was analyzed using the edgeR v3.24.2 package within R, applying the FDR control method to adjust p-values for multiple testing scenarios, assessing the significance of differential expression. For this research, genes exhibiting an adjusted p-value < 0.05 and an absolute log2 fold change (|log2FC|) ≥ 1 were selected for further analysis. Gene annotation was sourced from the Ensembl genome browser database version 96 (available at http://www.ensembl.org/index.html). Gene functions within KEGG pathways were annotated using ClusterProfiler, which was also used for functional enrichment analysis. The entire process of RNA sequencing and its comprehensive analysis was conducted by GeneFund Biotech Co. Ltd.

### Statistical analysis

Comprehensive statistical analyses were conducted using the SPSS software (version 25.0, based in Chicago, IL, USA). Results are presented as mean values ± SEM. A portion of the data was standardized to mitigate the impact of extraneous variation. One-way analysis of variance (ANOVA) based on LSD (equal variances assumed) post hoc analysis or Dunnett's T3 (equal variances not assumed) approach was conducted to assess significant differences between two groups among three or more groups. The BMS scores, assessed at various time points, were analyzed using repeated-measures ANOVA and post-hoc comparisons made via LSD tests. *A p*-value of less than 0.05 indicated statistical significance.

## Results

### HB promotes neuroprotection and functional restoration after SCI

**Figure 1:**
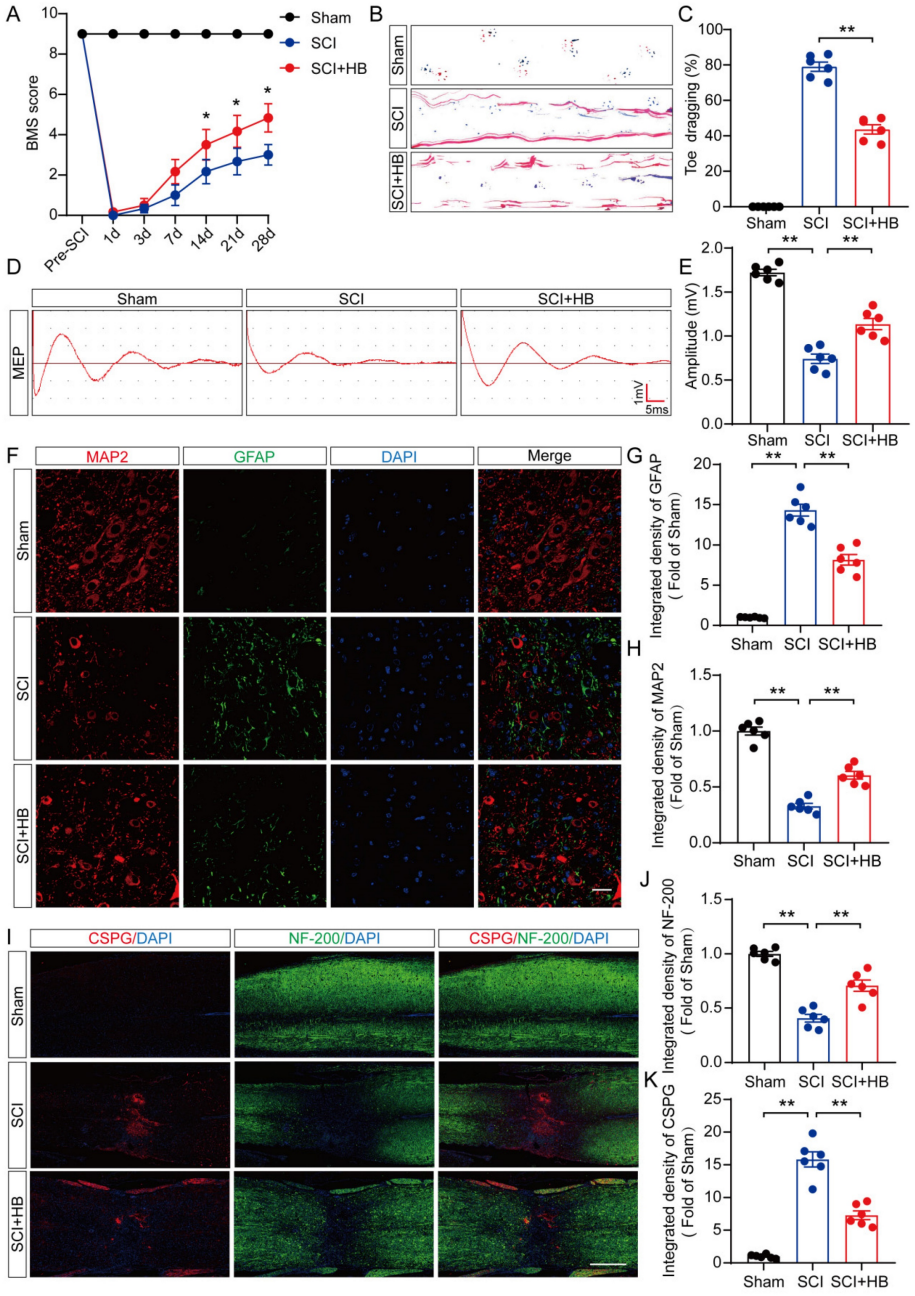
** HB improves functional restoration after SCI. (A)** BMS score of Sham, SCI, and SCI + HB group on days 1, 3, 7, 14, 21 and 28 after SCI. **(B)** Representative images of footprints used in walking analyses of mice on day 28 after SCI. Blue: forepaw print; Red: hindpaw print. **(C)** Toe dragging (%) analyses of mice on day 28 in the respective groups. **(D)** Representative diagrams of motor evoked potential (MEP) detection in 28 days after SCI from Sham, SCI, and SCI + HB group. **(E)** Quantitative analysis of the amplitude of the first peak (mV). **(F-H)** Representative images of immunofluorescent analysis of MAP2 (red) and GFAP (green) and DAPI (blue) in in sagittal sections of thoracic spinal cords on day 28 after SCI; scale bar: 100 μm. Quantitative analysis of MAP2 and GFAP immunofluorescence is shown in the graph on the right. **(I)** Representative images of immunofluorescence staining of CSPG (red) and NF-200 (green) in each group on day 28 after SCI; scale bar: 1,000 μm. **(J-K)** Quantitative analysis of the relative intergrated density of the fluorescence of NF-200 and CSPG. The data are presented as the means ± SEM (n = 6 mice per group); *P < 0.05, **P < 0.01.

In an initial exploration of HB's neuroprotective potential in mice, comprehensive experiments were conducted to assess its impact on recuperation following SCI, as depicted in **Figure S1A**. We monitored the effects of different doses of HB on the recovery of motor function in mice and found that the optimal therapeutic concentration of HB was 20 mg/kg **(Figure S1B)**. The BMS, an evaluative scale spanning 9 points, was used to quantify motor skill recovery and progression stages. Fourteen days post-treatment, the SCI + HB cohort demonstrated a notable enhancement in BMS scores relative to the SCI-only group **(Figure 1A, Figure S2A)**. This improvement was confirmed by footprint analyses and electrophysiological assessments, indicating diminished toe dragging and augmented amplitudes of MEPs in the HB-treated group **(Figures 1B-E, Figures S2B-E)**. To evaluate the safety and potential toxicity of HB in the long-term application, we preserved the heart, liver, spleen, lungs, and kidneys of the Sham group, SCI group, and SCI+HB group 56 days after injury. HE staining analysis of heart, liver, spleen, lung, and kidney tissue sections showed that no obvious abnormal signs were found in the SCI + HB group compared with the SCI group and the Sham group, proving that HB has good biological safety **(Figure S2F)**. Histologically, a comparison revealed that the area of glial scarring was significantly reduced, and there was an uptick in the count of motor neurons and SYN-positive synapses in the SCI + HB group compared to the SCI group, as shown in **Figures S1C-H**. The recuperation of the urinary system, a pivotal indicator of therapeutic success 32, was evidenced by the reduced bladder wall thickness in SCI mice treated with HB, suggesting an accelerated functional recovery of the bladder in the SCI + HB group, highlighted in **Figures S1I-J**. Subsequent examination of the axon density and condition within the lesion core sought to unravel the anatomical underpinnings of functional recovery. Immunofluorescence assays for MAP2 and GFAP demonstrated an increased density of MAP2-positive axons and a moderated expression of GFAP in the SCI + HB group in comparison to the SCI group, as presented in **Figures 1F-H**. Further exploration into nerve regeneration, through immunostaining for NF-200 (neurofilament) and CSPGs, identified as key inhibitory components for axon regeneration post-SCI, revealed these proteoglycans contribute to the barrier against axonal regrowth within the glial scar and perineuronal net 33, 34. Post-HB treatment, a significant upsurge in NF-200 intensity near the trauma site and a marked reduction in CSPG expression were observed, indicative of HB's facilitation of axonal regeneration and sprouting **(Figures 1I-K)**. Therefore, HB significantly promotes functional recovery post-SCI.

### HB attenuates pyroptosis and oxidative stress after SCI

**Figure 2:**
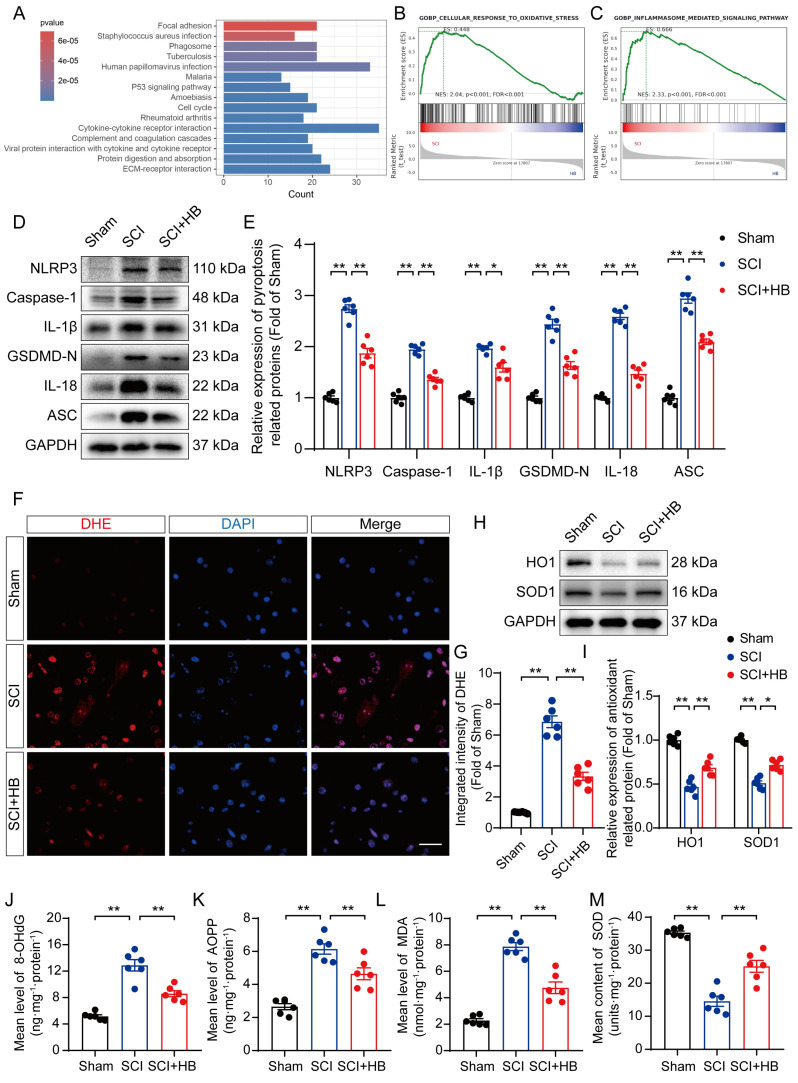
** HB attenuates pyroptosis and oxidative stress after SCI. (A)** The top 15 KEGG enrichment results of differentially expressed up-regulated genes between the SCI group and the SCI + HB group (n = 5). **(B-C)** GSEA analysis of differentially expressed pathway between the SCI group and the SCI + HB group enriched by KEGG. **(D-E)** WB analysis and quantification of NLRP3, caspase-1, IL-1β, GSDMD-N, IL-18 and ASC protein levels in spinal cord lesions from the indicated groups on day 3 after SCI. GAPDH was utilized as a loading control. **(F)** Frozen sections of spinal cord from each group were stained with DHE (red; a ROS fluorescent probe). DAPI staining for nuclei (blue; scale bar: 20 μm). **(G)** A quantification graph for DHE immunofluorescence from Sham, SCI, and SCI + HB group. **(H-I)** WB analysis and quantification of HO1 and SOD1 protein levels in spinal cord lesions from the indicated groups on day 3 after SCI. GAPDH was utilized as a loading control. **(J-L)** The quantities of 8-OHdG, AOPP and MDA in the injured spinal cord segments were detected by ELISA. **(M)** The enzyme content of SOD in the injured spinal cord segments were detected by ELISA. The data are presented as the means ± SEM (n = 6 mice per group); *P < 0.05, **P < 0.01.

Transcriptome analysis was carried out on the SCI and SCI + HB groups to delve into the molecular mechanisms through which HB exerts its effects; compared to the SCI group, there was an upregulation of the top 15 pathways in the KEGG enrichment analysis, notably including the "phagosome" pathway, as depicted in **Figure 2A.** Gene set enrichment analysis (GSEA) further indicated a reduction in the expression levels of genes associated with oxidative stress and pyroptosis following HB treatment, as shown in **Figures 2B-C**. WB analysis confirmed these findings, showing a significant decrease in the levels of proteins related to pyroptosis, such as NLRP3, caspase-1, GSDMD-N, IL-1β, IL-18, and ASC in the SCI + HB group compared to the SCI group, as presented in **Figures 2C-D**. IF staining also demonstrated that the expression levels of Caspase-1 and GSDMD-N in neurons were significantly lower in the SCI + HB group than in the SCI group, with a concurrent reduction in the numbers of caspase-1- and GSDMD-N-positive microglial cells, as illustrated in **Figures S3A-H**. The results from ELISA corroborated these observations, indicating lower concentrations of caspase-1, GSDMD-N, cleaved IL-1β, and cleaved IL-18 in the SCI + HB group, as shown in **Figures S3I-L**. Additionally, the study assessed oxidative stress levels across the groups. Using DHE as a ROS probe, a diminished level of oxidative damage was detected in the damaged spinal cord of the SCI + HB group, evidenced by weaker fluorescence signals, as indicated in **Figures 2F-G**. Western blot analysis for HO1 and SOD1 revealed elevated levels of these antioxidative proteins in the SCI+HB group, as depicted in **Figures 2H-I**. Moreover, ELISA results showed a significant reduction in the levels of 8-OHdG, AOPP, and MDA, alongside an upregulation of SOD content in the SCI + HB group, as presented in **Figures 2J-M**. Therefore, HB effectively mitigates pyroptosis and oxidative stress in the injured spinal cords.

### HB reinforces autophagy activity after SCI

**Figure 3:**
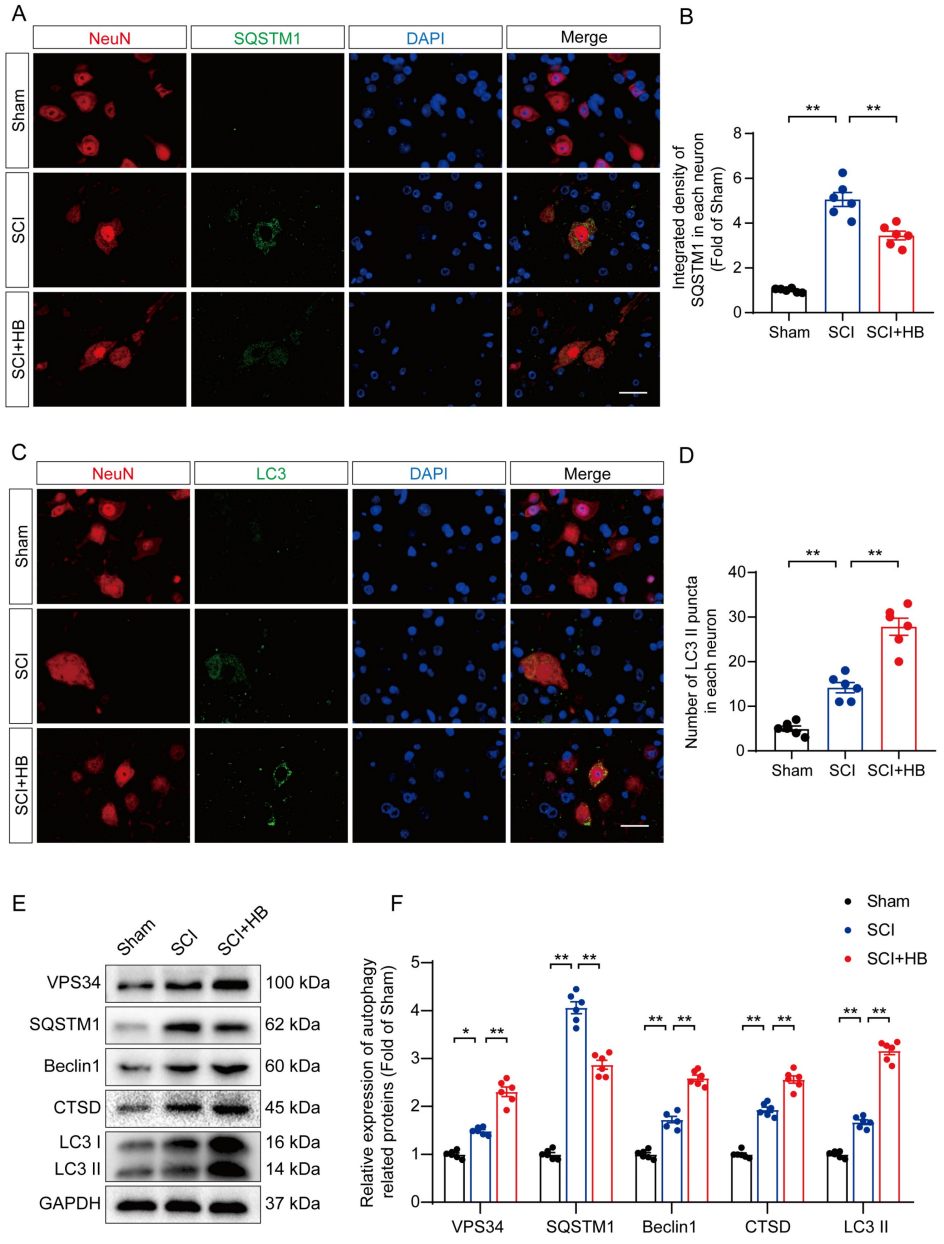
**HB enhances autophagy after SCI. (A-B)** Double immunofluorescence staining for SQSTM1 and NeuN in the spinal cord ventral horn grey matter from the groups on day 3 after SCI; scale bar: 20 μm. Quantification of SQSTM1 immunofluorescence staining is presented on the right of the representative images. **(C-D)** Double immunofluorescence staining showing LC3 and NeuN colocalization in the spinal cord ventral horn grey matter from all groups on day 3 after SCI; scale bar: 20 μm. The number of LC3 II puncta in each neuron is presented on the right. **(E-F)** WB analyses of VPS34, SQSTM1, Beclin1, CTSD and LC3 in the injured spinal cord lesion areas on day 3 after SCI. GAPDH was utilized as a loading control. The illustrations on the right present the summarized data from WB analyses. The data are presented as the means ± SEM (n = 6 mice per group); *P < 0.05, **P < 0.01.

Prior studies have identified autophagy as a pivotal pathway in the therapeutic intervention of CNS disorders, offering a protective shield against cell mortality provoked by neuroinflammation 35, 36. This investigation delved into HB's influence on autophagy subsequent to SCI, focusing on the modulation of proteins integral to autophagy. These proteins included autophagosome indicators (VPS34, Beclin1, LC3), the lysosomal protease CTSD, and the autophagy cargo receptor p62/SQSTM1. Immunofluorescence assays demonstrated a noticeable decrease in p62/SQSTM1 expression within neurons at the lesion epicenter after administering HB for three days post-SCI, compared to the control SCI group, as depicted in **Figures 3A-B**. Additionally, the observation of an augmented presence of LC3 II puncta within neurons signaled an enhancement in autophagosome accumulation post-SCI, with a further increase following HB administration, highlighted in **Figures 3C-D**. The quantification of autophagy-related proteins via Western blot analysis confirmed the upregulation of VPS34, Beclin-1, LC3 II, and CTSD post-HB treatment, while demonstrating a reduction in p62/SQSTM1 expression, as illustrated in **Figures 3E-F**. These observations underscore HB's capacity to elevate the expression of autophagy-associated lysosomal and autophagosomal markers, alongside diminishing the accumulation of autophagic substrates, thereby facilitating an improved autophagic flux in the aftermath of SCI.

### HB inhibits pyroptosis and oxidative stress by activating autophagy after SCI

**Figure 4:**
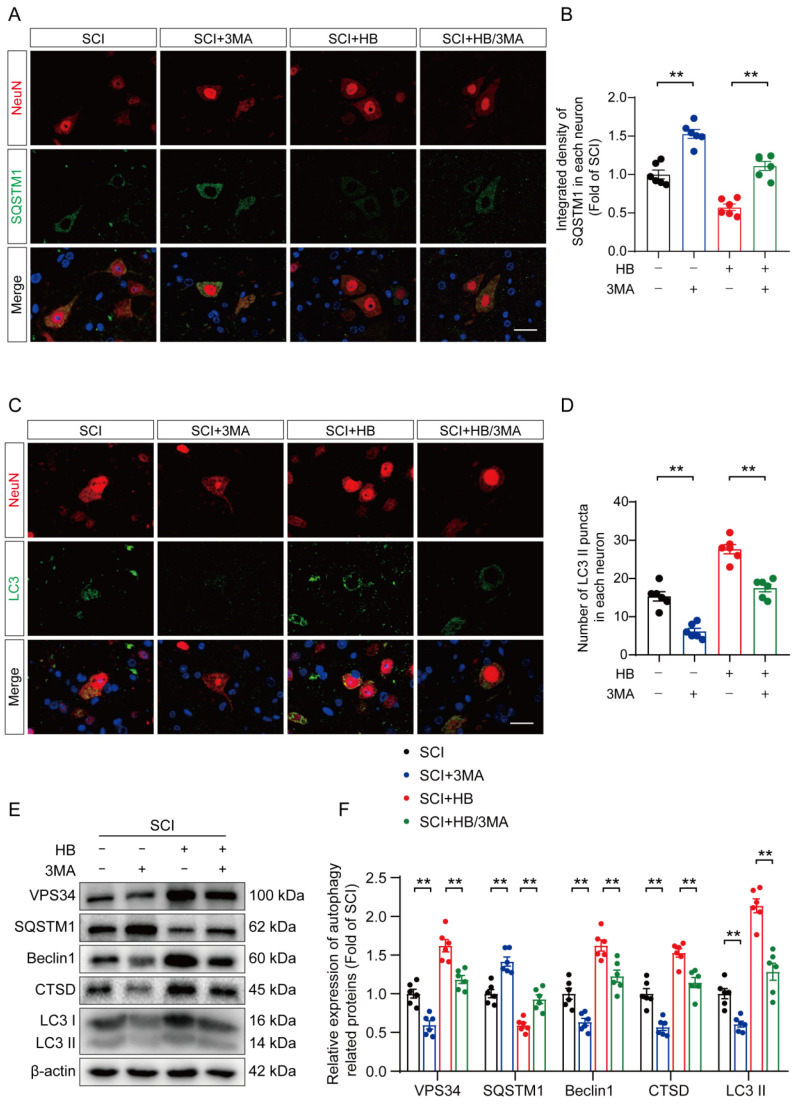
** 3MA counteracts autophagy enhancement attributed to HB after SCI. (A-B)** Double immunofluorescence staining for SQSTM1 and NeuN in the spinal cord ventral horn grey matter from the groups on day 3 after SCI; scale bar: 20 μm. Quantification of SQSTM1 immunofluorescence staining is presented on the right of the representative images.** (C-D)** Double immunofluorescence staining showing LC3 and NeuN colocalization in the spinal cord ventral horn grey matter from all groups (SCI, SCI + 3MA, SCI + HB, SCI + HB/3MA groups) on day 3 after SCI; scale bar: 20 μm. The number of LC3 II puncta in each neuron is presented on the right. **(E-F)** WB analyses of VPS34, SQSTM1, Beclin1, CTSD and LC3 in the injured spinal cord lesion areas on day 3 after SCI. β-actin was utilized as a loading control. The illustrations on the right present the summarized data from WB analyses. The data are presented as the means ± SEM (n = 6 mice per group); *P < 0.05, **P < 0.01.

To substantiate HB's therapeutic effect on enhancing autophagic activity in SCI recovery, we used 3MA. This targeted autophagy inhibitor inhibits the autophagy vesicle formation by inhibiting the PI3K/AKT/mTOR pathway alongside HB administration 37. This approach aimed to elucidate the interplay among autophagy, oxidative stress, and pyroptosis in an SCI model treated with HB. Illustrations in **Figures 4A-D** indicate that the SCI + HB/3MA cohort displayed a higher integrated density of p62/SQSTM1 and a reduced count of LC3 II puncta compared to the SCI + HB group. Correspondingly, Western blot analysis indicated a decrease in the levels of autophagy-related proteins VPS34, Beclin1, CTSD, and LC3 II, alongside an elevation in p62/SQSTM1 levels in the SCI + HB/3MA group when contrasted with the SCI + HB group, as shown in **Figures 4E-F**. These observations confirm that the concurrent administration of 3MA with HB notably obstructs autophagic activation.

**Figure 5:**
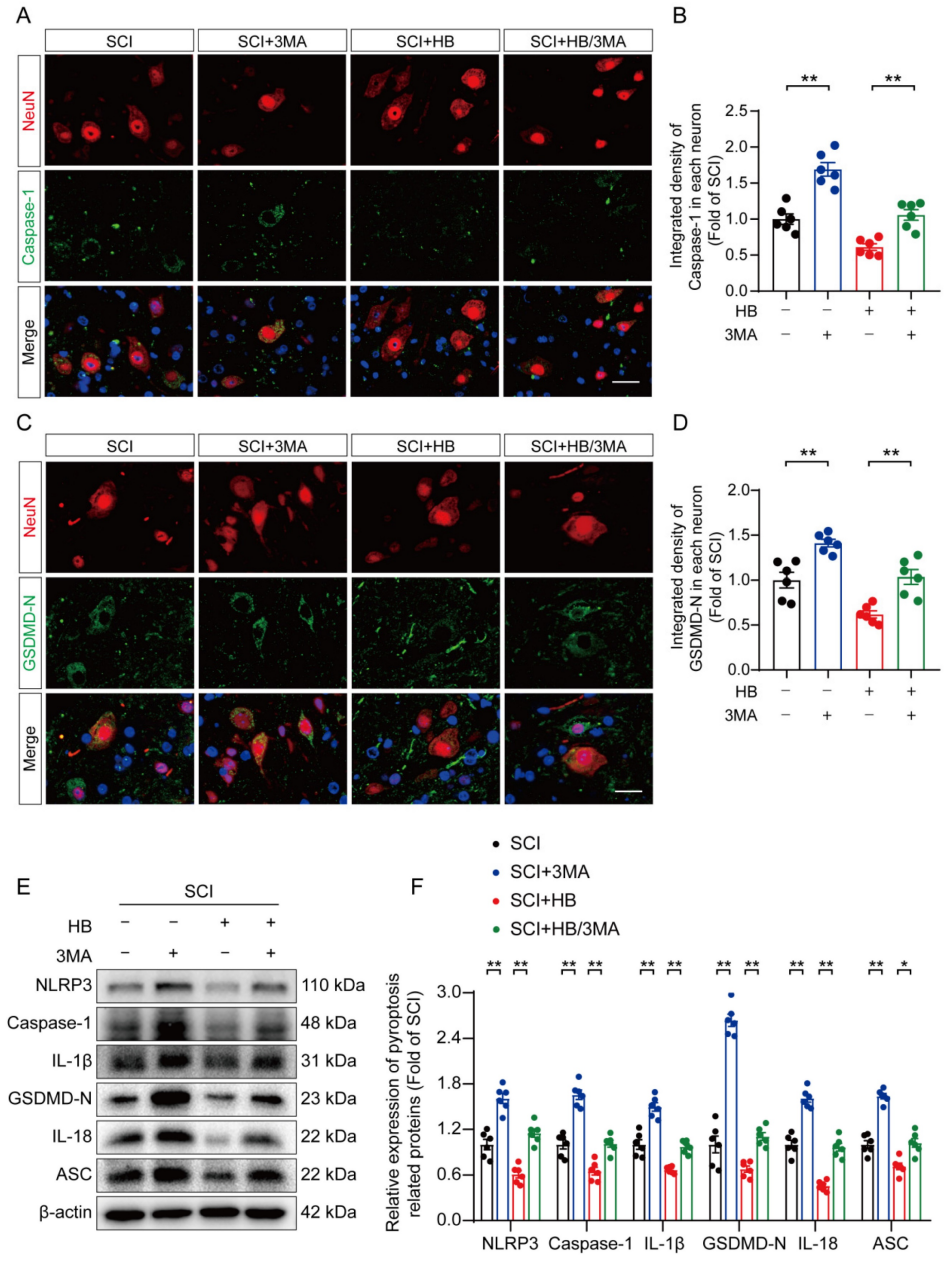
** HB inhibits pyroptosis by activating autophagy after SCI. (A-B)** Double immunofluorescence staining for Caspase-1 and NeuN in the spinal cord ventral horn grey matter from the groups on day 3 after SCI; scale bar: 20 μm. Quantification of Caspase-1 immunofluorescence staining is presented on the right of the representative images. **(C-D)** Immunofluorescence staining images of GSDMD-N and NeuN in spinal cords of the SCI, SCI + 3MA, SCI + HB and SCI + HB/3MA groups; scale bar: 20 μm. Quantified integrated intensity of GSDMD-N in neurons is presented on the right. (E-F) WB analysis and quantification of NLRP3, caspase-1, IL-1β, GSDMD-N, IL-18 and ASC protein levels in spinal cord lesions from the indicated groups on day 3 after SCI. β-actin was utilized as a loading control. The data are presented as the means ± SEM (n = 6 mice per group); *P < 0.05, **P < 0.01.

The influence of 3MA on pyroptosis and oxidative stress was examined to explore whether autophagy primarily facilitates neuronal recovery post-SCI through HB's action. IF staining shows an elevation in the fluorescence-integrated densities of caspase-1 and GSDMD-N in neurons, along with a rise in caspase-1- and GSDMD-N-positive microglial cells in the SCI + HB/3MA group, compared to the SCI + HB group three days post-injury, as shown in **Figures 5A-D and S4A-D**. Correspondingly, Western blot analysis revealed that the expression levels of proteins associated with pyroptosis were higher in the SCI + HB/3MA group, as depicted in **Figures 5E-F**. ELISA results confirmed that 3MA treatment significantly increased the levels of caspase-1, GSDMD-N, cleaved IL-1β, and cleaved IL-18 in the SCI + HB/3MA group in comparison with the SCI + HB group, as presented in **Figures S4E-H**. Additionally, the effect of autophagy inhibition on oxidative stress was assessed. WB analysis showed a reduction in antioxidant-related protein expression in the SCI + HB/3MA group compared to the SCI + HB group, illustrated in **Figures S5A-B**. DHE staining revealed more severe oxidative damage to nerve cells in spinal cord slices from the SCI + HB/3MA group, as seen in **Figures S5C-D**. Further ELISA findings indicated that 3MA treatment markedly raised the levels of 8-OHdG, AOPP, and MDA in the SCI + HB/3MA group while decreasing superoxide dismutase (SOD) content, as detailed in **Figures S5E-H**. These results imply that 3MA partially counteracts HB's suppressive effects on pyroptosis and oxidative stress.

### HB promotes functional recovery by upregulating autophagy after SCI

**Figure 6:**
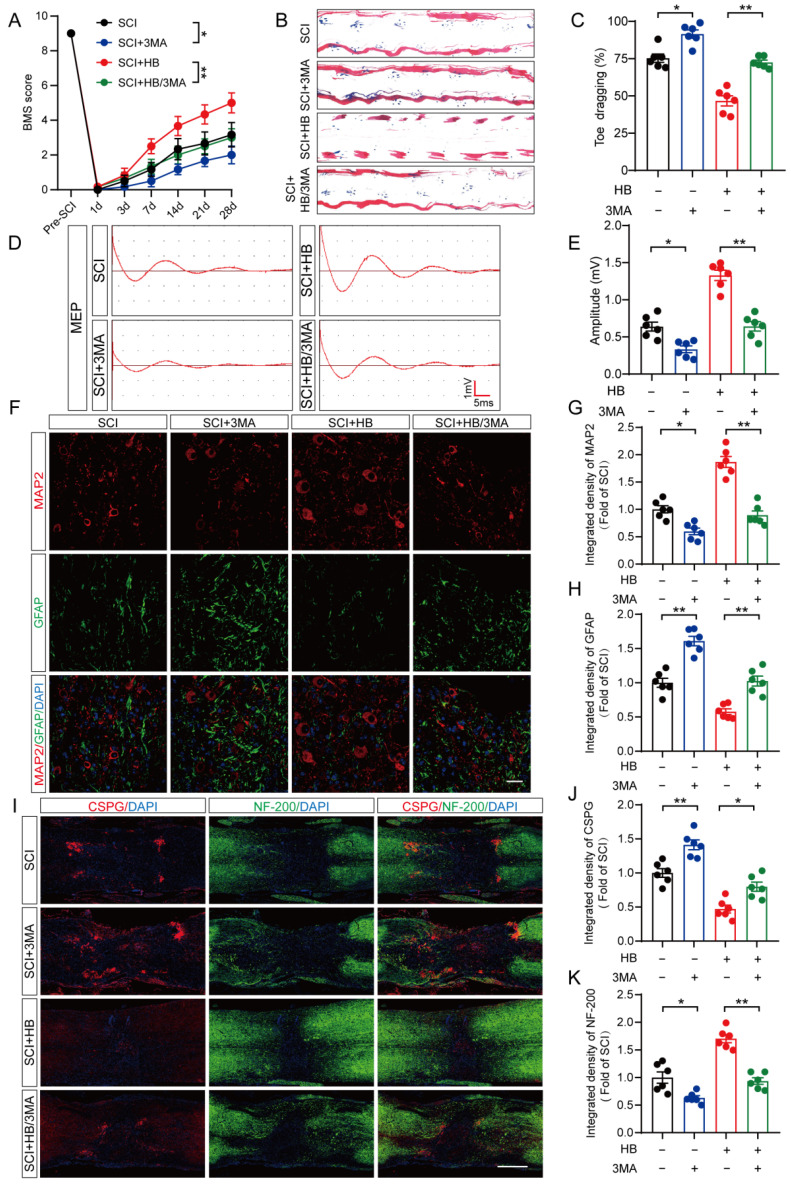
** HB enhances functional recovery by promoting autophagy. (A)** BMS score of SCI, SCI + 3MA, SCI + HB, and SCI + HB/3MA group on days 1, 3, 7, 14, 21 and 28 after SCI. **(B)** Representative images of footprints used in walking analyses of mice on day 28 after SCI. Blue: forepaw print; Red: hindpaw print. **(C)** Toe dragging (%) analyses of mice on day 28 in the respective groups. **(D)** Representative diagrams of motor evoked potential (MEP) detection in 28 days after SCI from SCI, SCI + 3MA, SCI + HB, and SCI + HB/3MA group. **(E)** Quantitative analysis of the amplitude of the first peak (mV). **(F-H)** Representative images of immunofluorescent analysis of MAP2 (red) and GFAP (green) and DAPI (blue) in in sagittal sections of thoracic spinal cords on day 28 after SCI; scale bar: 100 μm. Quantitative analysis of MAP2 and GFAP immunofluorescence is shown in the graph on the right. **(I)** Representative images of immunofluorescence staining of CSPG (red) and NF-200 (green) in each group on day 28 after SCI; scale bar: 1,000 μm. **(J-K)** Quantitative analysis of the relative integrated density of the fluorescence of NF-200 and CSPG. The data are presented as the means ± SEM (n = 6 mice per group); *P < 0.05, **P < 0.01.

Subsequent analyses on the damaged spinal cord tissues from all experimental groups were conducted, focusing on histomorphology and functional outcomes. On the 28th day following SCI, the BMS scores indicated a diminished motor capacity in the group treated with SCI + HB/3MA compared to the SCI + HB group, as shown in **Figure 6A**. Footprint analysis and electrophysiological assessments further revealed an increase in toe dragging and a decrease in MEPs amplitude in the SCI + HB/3MA group relative to the SCI + HB group, detailed in **Figures 6B-E**. Histological examinations utilizing H&E, Masson, and Nissl staining corroborated these outcomes, unveiling an expansion in the glial scar area and a decrease in the count of motor neurons in the SCI + HB/3MA group compared to the SCI + HB group, as evidenced in **Figures S6A-D**. A notable reduction in SYN-positive synapses was also observed in the SCI + HB/3MA group, highlighted in **Figures S6E-F**. Moreover, H&E staining of the bladder tissue illustrated a significant thickening of the bladder wall and disorganization of muscle fascicles in the SCI mice of the SCI + HB/3MA group compared to those in the SCI + HB group, as depicted in **Figures S6G-H**. The combined administration of 3MA and HB resulted in a disordered lesion area, a significant reduction in MAP2-positive axons, increased deposition of CSPGs, and decreased intensity of NF-200 positivity, aligning with a compromised neurological function recovery 28 days post-SCI **(Figures 6F-K)**. Therefore, HB facilitates functional recovery following SCI by augmenting autophagic activity.

### HB enhances autophagy and suppresses pyroptosis and oxidative stress by upregulating TFEB activity after SCI

The MiTF/TFE family of transcription factors, including MITF, TFEB, and TFE3, are global regulators of cell survival and energy metabolism through the promotion of lysosomal and anti-oxidative genes 38.

**Figure 7:**
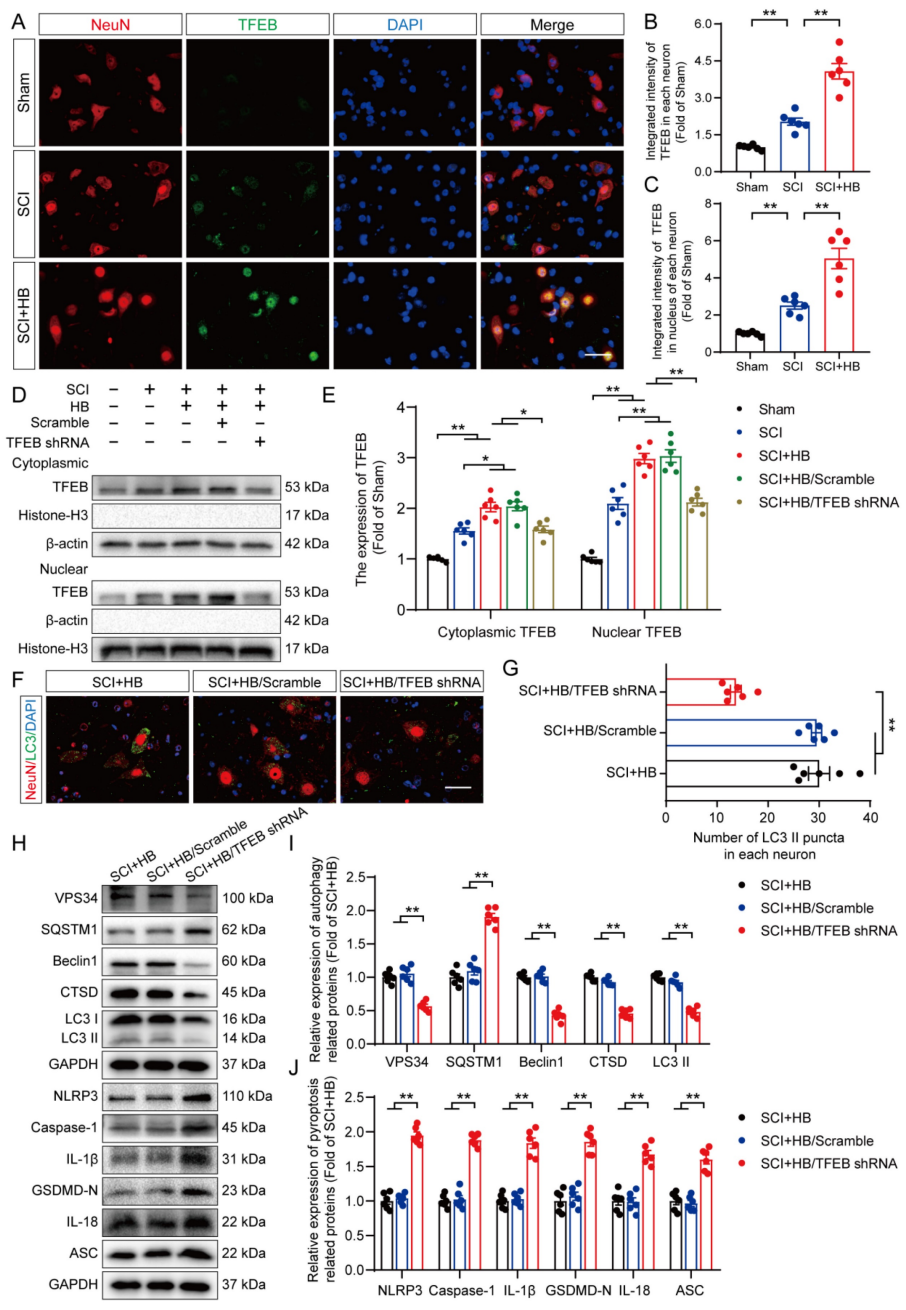
** HB promotes autophagy by increasing TFEB expression levels after SCI. (A)** Representative images of immunofluorescence staining for TFEB (green) and NeuN (red) in the injured spinal cord ventral horn grey matter on day 3 after SCI.** (B-C)** The illustration showing the quantitative results indicates that HB increased the integrated density of TFEB in spinal cord neurons and the integrated density of TFEB in the nucleus of each neuron. **(D-E)** WB analysis of cytoplasmic and nuclear TFEB levels in the injured spinal cord lesions on day 3 after SCI in each group (Sham, SCI, SCI + HB, SCI + HB/scrambled shRNA, and SCI + HB/TFEB shRNA groups). The data were normalized to β-actin or histone H3. **(F-G)** Typical immunofluorescence staining images of spinal cord ventral horn grey matter on day 3 after SCI, with the number of LC3 II-positive puncta in neurons shown in the graph; scale bar: 20 μm. **(H-J)** WB analysis of VPS34, SQSTM1, Beclin1, CTSD, LC3, NLRP3, caspase-1, IL-1β, GSDMD-N, IL-18 and ASC levels in injured spinal cord lesions on day 3 after SCI. GAPDH was utilized as a loading control. Densitometry quantification of the expression of autophagy- and pyroptosis-related proteins in the injured spinal cord lesions. The data are presented as the means ± SEM (n = 6 mice per group); *P < 0.05, **P < 0.01.

Our results showed differences in the mRNA levels of *Mitf, Tfeb*, and *Tfe3* after HB treatment. Specifically, after SCI, the mRNA levels of TFEB were significantly increased after HB treatment, while the mRNA levels of TFE3 and MITF did not change significantly **(Figures S7A-C)**. We also detected the mRNA levels of Nuclear erythroid 2-related factor 2 (Nrf2), a transcription factor that plays a key role in the regulation of inflammation 39. Interestingly, the mRNA level of *Nrf2* was significantly upregulated by HB treatment, indicating that Nrf2 may be another target of HB to inhibit oxidative stress **(Figures S7D)**. Subsequent basic research needs to be improved further. Thus, the present study delved into TFEB's activity within SCI models to understand how HB influences TFEB's regulation. Analytical results, as illustrated in **Figures 7A-C**, revealed that neurons in the SCI + HB group showed a substantial increase in TFEB activity within both the cytoplasm and nucleus compared to the SCI group. To delve deeper into how HB activates TFEB to bolster autophagy and simultaneously diminish pyroptosis and oxidative stress, we utilized adeno-associated virus (AAV)-TFEB short hairpin RNA (shRNA) to downregulate TFEB expression. This approach facilitated a comparative analysis across the Sham, SCI, SCI + HB, SCI + HB/scrambled shRNA, and SCI + HB/TFEB shRNA groups. Western blot findings indicated a pronounced reduction in TFEB presence in the cytoplasm and nucleus in the SCI + HB/TFEB shRNA group when contrasted with the SCI + HB/scrambled shRNA group. No significant alterations were detected between the SCI + HB and SCI + HB/scrambled shRNA groups, as evidenced in **Figures 7D-E**. This data underscores that TFEB shRNA transfection successfully suppressed TFEB expression and its relocation to the nucleus following SCI.

Subsequently, we evaluated whether HB-induced nuclear translocation of TFEB plays a role in modulating autophagy, pyroptosis, and oxidative stress. IF analysis demonstrated that the quantity of LC3 II positive puncta within neurons of the SCI + HB and SCI + HB/scrambled shRNA groups showed no significant variance; however, a considerable reduction in LC3 II positive puncta was observed in neurons of the SCI + HB/TFEB shRNA group, as illustrated in **Figures 7F-G**. WB analyses further supported these findings, indicating no significant change in the expression levels of autophagy-related proteins (VPS34, SQSTM1, Beclin1, CTSD, and LC3) between the SCI + HB and SCI + HB/scrambled shRNA groups. In contrast, in the SCI + HB/TFEB shRNA group, there was a notable decrease in the expression of VPS34, Beclin1, CTSD, and LC3 II, with an increase in SQSTM1 expression, as shown in **Figures 7H-I**. Moreover, the introduction of AAV-TFEB shRNA reversed HB's impact on pyroptosis and oxidative stress following SCI, as evidenced in **Figures 7H, J, and S8A-D**. These observations suggest that the stimulation and nuclear translocation of TFEB are key mechanisms through which HB elevates autophagic activity and diminishes pyroptosis and oxidative stress post-SCI.

### HB stimulates TFEB activity via the AMPK-TRPML1-calcineurin signaling pathway after SCI

**Figure 8:**
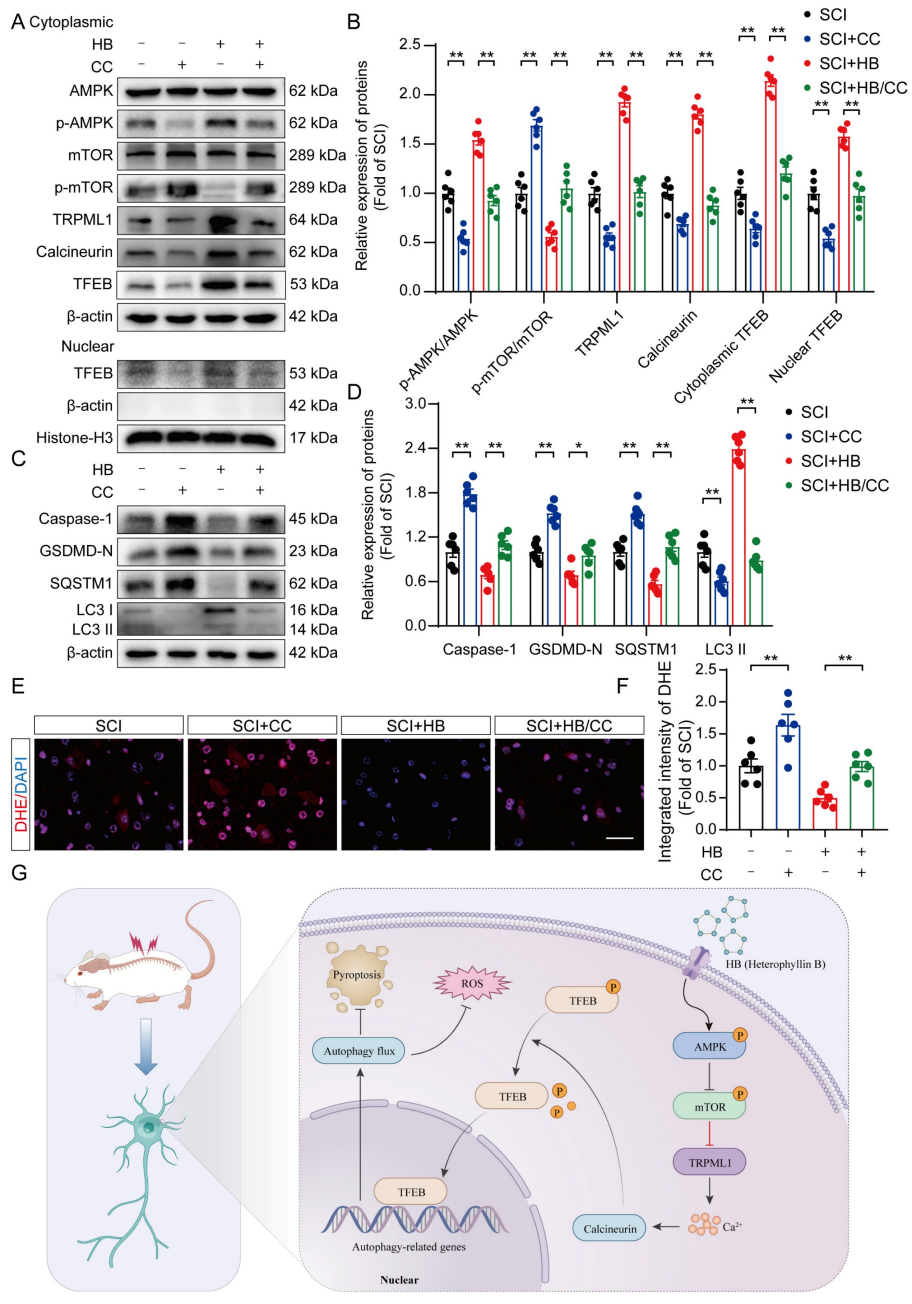
** HB activates TFEB through the AMPK-TRPML1-calcineurin signaling pathway. (A-D)** WB analysis of AMPK, p-AMPK, mTOR, p-mTOR, TRPML1, calcineurin, cytoplasmic and nuclear TFEB, caspase-1, GSDMD-N, SQSTM1, LC3 expression levels in the SCI, SCI + CC, SCI + HB and SCI + HB/CC groups. β-actin and Histone-H3 was used as a loading control. The densitometry quantification shown on the right reveals that CC inhibited the HB-induced effects. **(E)** Frozen sections of spinal cord from each group were stained with DHE (red; a ROS fluorescent probe). DAPI staining for nuclei (blue; scale bar: 20 μm). **(F)** A quantification graph for DHE immunofluorescence from SCI, SCI + CC, SCI + HB and SCI + HB/CC group. **(G)** Schematic illustrating the proposed molecular mechanism by which HB enhances autophagy after SCI by activating the AMPK-TRPML1-calcineurin signaling pathway, thereby promoting the nuclear translocation of TFEB. The induction of autophagy inhibits pyroptosis and oxidative stress progression in the injured spinal cord. The data are presented as the means ± SEM (n = 6 mice per group); *P < 0.05, **P < 0.01.

Earlier investigations have illuminated the AMPK-TRPML1-calcineurin signaling pathway as a crucial calcium-mediated regulatory mechanism affecting the MiT/TFE family's activity 40, 41. This finding suggests HB's potential modulation via calcium-dependent signaling mechanisms. In dissecting this pathway's role, particularly in the context of SCI treated with HB, we meticulously separated cytoplasmic proteins from nuclear proteins to scrutinize the expression profiles of key proteins in this pathway. WB analysis revealed that post-HB application, the cytoplasmic expressions of AMPK and mTOR remained stable. However, a notable increase in p-AMPK alongside a reduction in p-mTOR was observed, as detailed in **Figures 8A-B**. Significantly, activities of the downstream signaling molecules TRPML1 and calcineurin showed a marked increase. Concurrently, HB was found to significantly elevate Transcription Factor EB (TFEB) within both the cytoplasm and nucleus, as indicated in **Figures 8A-B**. These findings underscore that HB engages the AMPK-TRPML1-calcineurin pathway, alongside TFEB, suggesting a sophisticated mechanism of action involving calcium signaling in promoting autophagic regulation and neuroprotection in SCI models.

To further explore whether the activation of TFEB following HB treatment is mediated through the AMPK-TRPML1-calcineurin signaling pathway, we examined the influence of CC (a known AMPK inhibitor), MHY1485 (an mTOR agonist), and tacrolimus (a calcineurin inhibitor) on this pathway. Illustrated in **Figures 8A-B**, the levels of phosphorylated AMPK (p-AMPK) in the SCI + HB/CC group were notably lower than in the SCI + HB group.

Additionally, there was an upsurge in the expression of p-mTOR among downstream molecules, while the expression levels of TRPML1 and calcineurin were significantly reduced **(Figures 8A-B)**. Western blot analysis further confirmed that, relative to the SCI + HB group, TFEB levels in both the cytoplasm and nucleus were significantly diminished in the SCI + HB/CC group **(Figures 8A-B)**. Moreover, we assessed the involvement of the AMPK-TRPML1-calcineurin pathway in HB's modulation of pyroptosis, autophagy, and oxidative stress. Western blot assays indicated that CC injection counteracted HB's effects on autophagy and pyroptosis in the SCI + HB/CC group **(Figures 8C-D)**. Staining with dihydroethidium indicated a significant intensification of oxidative harm in the spinal cord after administering CC in the group with SCI + HB/CC** (Figures 8E-F)**. Consistently, the effects of MHY1485 and tacrolimus were also significantly reversed by HB **(Figures S9A-D)**. To conclude, the evidence supports that HB enhances TFEB expression through the AMPK-TRPML1-calcineurin signaling cascade in cases of SCI.

## Discussion

Traumatic SCI represents a profound challenge for individuals and the broader healthcare system, imposing significant socioeconomic strains 2. Despite concerted efforts spanning several decades to identify novel therapeutic targets through preclinical and clinical research, an efficacious and practical clinical therapy for traumatic SCI has yet to be realized 5, 42. The delineation between the irreversible damage of primary injuries and the potentially reversible nature of secondary injuries has emerged as a significant milestone in SCI research. The latter, characterized by modifiable pathological processes, opens avenues for therapeutic interventions. The scientific consensus underscores the importance of attenuating secondary damage, particularly by curbing the inflammation that ensues post-SCI, as a pivotal strategy for enhancing patient outcomes 43. The processes of neuroinflammation and cellular demise that follow SCI critically impact neurons and glial cells, marking secondary injury mechanisms 44. A promising therapeutic approach involves mitigating early neuroinflammation to curb inflammatory neuronal death during this secondary injury phase 8, 45. Given this framework, the anti-inflammatory capabilities of HB emerge as a potentially valuable intervention for early SCI treatment. This research pioneers in demonstrating the therapeutic efficacy of HB for SCI, delving into its mechanisms of action in mitigating oxidative stress and pyroptosis by promoting autophagy through TFEB activation.

Renowned in traditional Chinese medicine, *Pseudostellaria heterophylla* has been utilized to address conditions such as anorexia, fatigue, weakness of the spleen, and heart palpitations. This herb, linked with the spleen meridian, is acknowledged for its immune-boosting properties 46. Following damage to the central nervous system (CNS), neuroinflammation occurs, triggered by the innate immune response. This inflammatory process involves cytokines and chemokines produced by astrocytes, microglia, endothelial cells, and immune cells from the periphery 8. In the context of SCI, the immune response is instrumental in facilitating nerve repair. Among the compounds derived from *Pseudostellaria heterophylla*, HB stands out due to its favorable physicochemical attributes and minimal toxicity 25. Given its potency against tumors and inflammatory conditions, HB has garnered interest. Research indicates HB's efficacy in reducing inflammation, fostering axonal growth, and offering neuroprotection in Alzheimer's disease scenarios 27, 28. Yet, its application in SCI treatment remains unexplored. This study used a spinal cord contusion model in mice to assess HB's impact on CNS trauma and showed that HB promoted axonal growth, diminished CSPG accumulation, and notably decrease the area of glial scarring in the damaged spinal cord. Analyses of footprints and electrophysiological assessments pointed to significant enhancements in the hind limb functionality of mice treated with HB. Post-SCI conditions often lead to prolonged difficulties in urination and urine retention, causing the bladder wall to thicken due to an increased collagen-to-muscle ratio 47. Histological examination with H&E staining demonstrated notable improvements in bladder tissue functionality following HB intervention. This study pioneers in documenting HB's role in augmenting functional recovery post-SCI in mice.

Inflammasomes are typically structured around three key components: a sensor that recognizes cytosolic patterns, an inflammatory caspase enzyme, and an adaptor molecule known as ASC, which orchestrates the interaction between the sensor and caspase 48. Pyroptosis, a form of programmed cell death, is marked by the hyperactivation of the NLRP3 inflammasome, initiating the creation of pores in cell membranes, leading to cell swelling, rupture, and the eventual expulsion of cellular components 13, 49. By finely tuning the regulation of pyroptosis, it might be possible to avert excessive loss of neurons 50. In pursuit of this, we utilized advanced molecular methodologies to explore the potential of HB in curbing pyroptosis.

The WB and enzyme-linked immunosorbent assay evidence confirmed that HB significantly curtailed the levels of proteins linked to pyroptosis in the aftermath of SCI. It has been well-documented that microglia are predominantly implicated in pyroptosis within the context of neuroimmune disorders of the CNS 51, 52. To ascertain the impact of HB on pyroptosis, we measured the expression of related markers in neurons and microglial cells via immunofluorescence staining, which unveiled HB's efficacy in dampening pyroptosis across these cellular types. The role of escalated ROS-induced oxidative stress in exacerbating secondary injury following SCI is well-established 53. The generation of oxidative stress and ROS is indispensable for the activation of the NLRP3 inflammasome, which in turn triggers pyroptosis through upregulation of NLRP3, pro-caspase-1, and pro-IL-1β 54. Our findings suggest that HB significantly mitigates oxidative stress subsequent to SCI. The transcription factor Nrf2 plays a pivotal role in orchestrating the cellular defense against oxidative stress, enhancing the resilience of neuronal cells to oxidative challenges, and safeguarding neurological functions 55. Future research is required to elucidate whether the beneficial effects of HB on functional recovery post-SCI are mediated through a mechanism involving the inhibition of oxidative stress via Nrf2.

Autophagy is a crucial cellular mechanism that maintains equilibrium in healthy cells and protects against CNS pathologies and trauma 22, 35. In scenarios of traumatic SCI, autophagy emerges as a vital survival strategy, curbing neuronal loss and enhancing neuroprotective outcomes 56. Extensive research underscores the importance of preserving optimal autophagic flux during SCI, essential for eliminating damaged cellular components, organelles, and apoptotic cells, supporting neuroprotective strategies and facilitating recovery 56, 57. In this study, we discovered that HB activated autophagy, which mitigated oxidative stress and pyroptosis, indicating that the mechanism by which HB modulated these processes was likely through autophagic regulation. To validate this hypothesis, we employed the autophagy inhibitor 3MA and observed that blocking autophagy negated the therapeutic effects of HB. Hence, our findings introduced the novel concept that HB enhanced functional recovery post-SCI by upregulating autophagy, thereby controlling oxidative stress and pyroptosis.

This study aimed to elucidate the specific mechanism by which HB influences autophagy within the context of SCI. Notably, previous investigations have demonstrated that the enhancement of autophagy, driven by TFEB and facilitated through the activation of the AMPK signaling pathway, is pivotal for neurological recovery in models of SCI 50, 58. Thus, we pinpointed TFEB as a critical factor for in-depth analysis. AMPK's role as an essential regulator in the MITF family in triggering autophagy via the AMPK/mTOR signaling axis has received substantial validation 26, 30. The activation of AMPK/mTOR signaling has been demonstrated to be associated with autophagy activation 59. The lysosomal calcium channel TRPML1, known for its localization within lysosomes, plays a significant role in this regulatory process 60. It has been previously established that the suppression of mTOR activity alongside TRPML1-mediated calcium release from lysosomes initiates calcineurin activation. This process promotes the dephosphorylation of TFEB, culminating in its migration into the nucleus 61. In our investigations, HB's role in promoting recovery from SCI was attributed to its activation of the AMPK-TRPML1-calcineurin signaling pathway. Further, combined treatment with HB and the AMPK inhibitor CC inhibited TFEB's nuclear translocation and transcriptional activities, attenuating the autophagic and anti-inflammatory responses previously observed with HB treatment alone. Hence, this research uniquely contributes to the scientific understanding by demonstrating that HB facilitates TFEB's nuclear translocation through the AMPK-TRPML1-calcineurin pathway in an SCI model, marking a novel discovery in the field.

This study has limitations. Research has indicated that targeting the cGAS-STING pathway is also a promising strategy for treating CNS injuries 62. Additionally, studies have confirmed that HB has an inhibitory effect on STING 26. The regulation of STING by HB in SCI requires further investigation. However, more research on the pharmacological mechanisms of HB is required before initiating clinical translation. The oral route is a drug delivery route with high safety and good compliance and has thus gradually become the main route of administration. Based on the US Food and Drug Administration (FDA) industry guidance on selecting safe starting doses for clinical trials 63, the optimal murine dose of 20 mg/kg of HB utilized in this study is equivalent to the human dose of 0.11 g. HB is one of the active compounds extracted from the ethyl acetate extract of *Pseudostellaria heterophylla* and is often used as the quality control index for Radix Pseudostellariae recorded in the Chinese Pharmacopoeia 64. Some *in vivo* studies found that oral administration of 1.5 g/day of *Pseudostellaria heterophylla* was effective in treating type 2 diabetes 65, so the human equivalent dose of HB could be attained through human oral consumption. The specific human oral dosage needs further study in the future. Typically, the onset of traumatic SCI is unpredictable, and the condition progresses rapidly. It is, therefore, crucial to determine the optimal treatment framework for HB. Overall, more research is still needed to complete a comprehensive evaluation before clinical translation. Additionally, the potential influence of HB on various cell death processes, including ferroptosis, parthanatosis, and NETosis, which are intricately linked with neurodegenerative diseases and disorders, merits thorough investigation. The reliance on shRNA-mediated knockdown of TFEB in our initial studies necessitates further validation through comprehensive research utilizing knockout mouse models to substantiate these initial observations more robustly.

## Conclusions

Our research demonstrates that HB facilitates the movement of TFEB into the nucleus via the AMPK-TRPML1-calcineurin signaling pathway, significantly boosting autophagy in cases of SCI. This activity was instrumental in reducing pyroptosis and oxidative stress within the damaged spinal cord, aiding in the recovery of function post-SCI as illustrated in **Figure 8G**. This investigation stands as the inaugural study to offer compelling evidence of HB's advantageous impact on SCI therapy. The results broaden the scope of knowledge regarding HB's anti-inflammatory effects in SCI repair and herald new avenues for the clinical application and understanding of HB in the management of SCI.

## Supplementary Material

Supplementary methods, figures and table.

## Abbreviations

SCI: Spinal cord injury
CNS: Central nervous system
HB: Heterophyllin B
TFEB: Transcription factor EB
MAP2: Microtubule-associated protein 2
GFAP: Glial fibrillary acidic protein
CSPG: Chondroitin sulfate proteoglycan
NF-200: Neurofilament H
SYN: synaptophysin
NLRP3: NLR family pyrin domain containing 3
ASC: Apoptosis-associated speck-like protein containing a caspase recruitment segment
IL-1β: Interleukin 1β
IL-18: Interleukin 18
GSDMD: Gasdermin D
ROS: reactive oxygen species
SOD1: Superoxide dismutase 1
HO1: Heme oxygenase 1
8-OHdG: 8-hydroxy-2 deoxyguanosine
AOPP: advanced oxidation protein products
MDA: Malondialdehyde
LC3: Microtubule-associated protein 1 light chain 3
CTSD: Cathepsin D
WB: Western blotting
IF: Immunofluorescence
AAV: Adeno-associated virus
H&E: Haematoxylin-eosin
ELISA: Enzyme-linked immunosorbent assay
MEP: Motor evoked potential
BMS: Basso Mouse Scale
DHE: Dihydroethidium
3MA: 3-methyladenine
AMPK: Adenosine 5-monophosphate-activated protein kinase
CC: Compound C
mTOR: Mammalian target of rapamycin
TRPML1: Transient receptor potential mucolipin 1
LSD: least significant difference
SEM: standard error of the mean
