# Supplementary material for: Heterophyllin B enhances transcription factor EB-mediated autophagy and alleviates pyroptosis and oxidative stress after spinal cord injury
Source: Int J Biol Sci. 2024 Oct 7;20(14):5415–35. doi: 10.7150/ijbs.97669 (PMC11528460; doi:10.7150/ijbs.97669)
Supplement: Supplementary file 1 — Supplementary methods, figures and table. [file ijbsv20p5415s1.pdf]

Supplementary Information

Heterophyllin B enhances transcription factor EB-mediated autophagy and alleviates pyroptosis and oxidative stress after spinal cord injury

Haojie Zhang *et. Al*

Supplementary methods

Antibodies

Primary antibodies specific to MAP2 (catalog # ab32454), NeuN (catalog # ab177487), Iba-1 (catalog # ab283319), synaptophysin (SYN, catalog # ab32594), chondroitin sulfate proteoglycans (CSPG, CS-56, catalog # ab11570), cathepsin D (CTSD, catalog # ab75852), SQSTM1/p62 (catalog # ab56416), TRPML1/MG-2 (catalog # ab28508), and calcineurin (catalog # ab52761) were sourced from Abcam, located in Cambridge, UK. Cell Signaling Technology, based in Danvers, MA, USA, provided primary antibodies for ASC (catalog # 67824), NLRP3 (catalog # 15101), AMPK (catalog # 5832), phosphorylated AMPK (catalog # 2531), mTOR (catalog # 2983), phosphorylated mTOR (catalog # 5536), and Histone-H3 (catalog # 4499). Sigma-Aldrich, situated in St. Louis, MO, USA, supplied the primary antibody for LC3B (catalog # L7543). Proteintech Group, from Chicago, Illinois, USA, furnished primary antibodies against Beclin1 (catalog # 11306-1-AP), SOD1 (catalog # 10269-1-AP), HO1 (catalog # 10701-1-AP), VPS34 (catalog # 12452-1-AP), caspase-1 (catalog # 22915-1-AP), TFEB (catalog # 13372-1-AP), GAPDH (catalog # 10494-1-AP), and β-actin (catalog # 66009-1-Ig). Affinity Biosciences, Ohio, USA, was the source for the primary antibody against GSDMD (catalog # AF4012). ABClonal Technology, Cambridge, MA, USA, provided primary antibodies against IL-1β (catalog # A16288), Neurofilament H (NF-200; catalog # A8442), and IL-18 (catalog # A1115). Santa Cruz Biotechnology, based in Santa Cruz, CA, USA, supplied the primary antibody against GFAP (catalog # sc-33673). Abcam supplied secondary antibodies including goat anti-rabbit IgG H&L (DyLight® 488) preadsorbed (catalogue number ab96899), goat anti-rabbit IgG H&L (DyLight® 594) preadsorbed (catalogue number ab96901), goat anti-mouse IgG H&L (DyLight® 488) preadsorbed (catalogue number ab96879), and goat anti-mouse IgG H&L (DyLight® 594) preadsorbed (catalogue number ab96881). Additionally, HRP-conjugated AffiniPure goat anti-mouse IgG (H+L) (catalogue number SA00001-1) and HRP-conjugated AffiniPure goat anti-rabbit IgG (H+L) (catalogue number SA00001-2) were obtained from the Proteintech Group.

Tissue section preparation and H&E, Nissl and Masson staining

At 14 and 28 days post-procedure, mice underwent deep anesthesia once again, followed by intracardiac infusion with phosphate-buffered saline (PBS, pH 7.4). Subsequently, a solution of 4% paraformaldehyde (PFA) in PBS was utilized for perfusion of the specimens. Sections of the spinal cord, both a 1-mm length segment located 4 mm from the injury epicenter and a complete 10-mm segment centered around the epicenter, were isolated and immersed in 4% PFA for a 24-hour period for fixation. These specimens were then subjected to a sequential dehydration process using escalating concentrations of ethanol before being embedded in paraffin aligned properly for sectioning. Using a microtome, 5-μm thick sections of the paraffin-embedded spinal cord tissue were prepared and placed onto gelatin-coated slides. Histopathological evaluations of both transverse and longitudinal sections were performed after hematoxylin and eosin (HE) staining. Heart, liver, spleen, lung, and kidney tissues were also collected, and HE staining was performed at week 8 post-injury. For Nissl staining, transverse sections underwent treatment with 1% cresyl violet acetate, adhering to the manufacturer's protocol, to highlight Nissl-positive cells indicative of neurons within the anterior horn. Longitudinal sections were processed for Masson's trichrome staining by incubation in a solution containing 10% potassium dichromate and 10% trichloroacetic acid, followed by nuclear staining with hematoxylin. These sections were then differentiated with hydrochloric acid in ethanol, turned blue with diluted ammonia, and finally stained using Masson's trichrome solution. The areas stained by Masson's trichrome (blue) were quantified using the thresholding feature in ImageJ software.

Immunofluorescence staining (IF)

For the immunofluorescence (IF) examination, sections from the spinal cord, both in transverse and longitudinal orientations, were meticulously prepared at a uniform thickness of 5 micrometers, in alignment with the previously established protocol. Following this, sections derived from each group underwent a series of preparatory steps including deparaffinization to remove wax, rehydration to restore water content, and thorough washing. The sections were then immersed in a sodium citrate buffer

(concentration of 10.2 mM) and subjected to heat-induced antigen retrieval by warming to 95 °C for a duration of 20 minutes. Permeabilization was carried out with a 0.1% solution of PBS-Triton X-100 for 10 minutes to allow intracellular access. This step was followed by blocking non-specific binding sites with a 10% solution of goat serum in PBS for one hour. The primary antibodies targeted towards LC3B (1:200), SQSTM1/p62 (1:200), caspase-1 (1:200), GSDMD (1:200), TFEB (1:100), MAP2 (1:500), GFAP (1:200), CSPG (1:200), NF-200 (1:100), SYN (1:500), Iba-1 (1:200), and NeuN (1:500) were then applied to the sections and left to incubate overnight at 4 °C, ensuring optimal binding to their respective antigens. The next day, to facilitate the binding of secondary antibodies, sections were first brought to room temperature for one hour, then incubated with secondary antibodies at 37 °C for another hour. To finalize the staining process, DAPI was applied to counterstain the nuclei, enhancing the visualization of cellular structures under fluorescence microscopy.

IF quantification

Immunofluorescence staining targeted at MAP2, GFAP, CSPG, and NF-200 was meticulously conducted on longitudinal spinal cord sections. Visualization of the stained lesion areas was achieved through the advanced optics of a Zeiss LSM 800 confocal microscope. For image processing and presentation, Zen Blue software by Zeiss and ImageJ software (Version 1.52a) were employed, ensuring high-quality image analysis. Images of transverse sections were specifically captured within a 0.5–1 mm range rostral to the lesion site, maintaining consistency in imaging across samples. Imaging analysis was further refined using a high-resolution fluorescence microscope from Olympus, Japan. This step involved selecting six distinct areas for image capture within the anterior horn across three sections chosen at random from each sample, to ensure a comprehensive assessment. The analysis in ImageJ software allowed for the precise quantification of the integrated density of key proteins such as caspase-1, GSDMD-N, and SQSTM1/p62 in neuronal cells. Additionally, the enumeration of LC3 puncta within neurons and the counting of caspase-1 and

GSDMD-N positive microglia cells per 0.1 mm^2 were performed manually, adhering to a rigorous double-blind procedure to uphold the integrity of the data collection process.

Enzyme-linked immunosorbent assay (ELISA)

A spinal cord tissue section, measuring 1 cm and centered on the injury locus, underwent homogenization in phosphate-buffered saline. This process involved several cycles of rapid freezing using liquid nitrogen followed by thawing, ensuring complete breakdown of tissue structure. The homogenized sample was then centrifuged at 10,000 × g for a duration of 10 minutes at 4 °C, facilitating the collection of the supernatant, which was earmarked for subsequent analysis. The detection of molecular markers indicative of inflammation and oxidative stress, specifically the cleaved forms of IL-1β and IL-18, caspase-1, GSDMD, 8-OHdG, and AOPP, within the spinal cord's lesioned regions, was achieved through the utilization of ELISA kits. These kits were employed as per the instructions provided by Jinhengnuo Biotechnology, based in Hangzhou, China. The determination of the concentrations of these biomolecules involved measuring the optical densities of the assay samples at a primary wavelength of 550 nm and an adjustment wavelength of 450 nm, using a microplate reader designed for such analyses.

Real-time quantitative PCR

Total RNA was obtained from the spinal cord using TRIzol reagent according to the manufacturer’s instructions. A two-step reaction process was performed: reverse transcription (RT) and PCR. The expression levels of targeted mRNAs were normalized to *β-Actin* mRNA expression. The $2^{-\Delta\Delta Ct}$ algorithm was used for evaluating relative expression levels. The primer sequences were designed and synthesized by GeneChem based on mRNA sequences obtained from the NCBI database. The primer sequences are listed in Table S1.

Figure S1

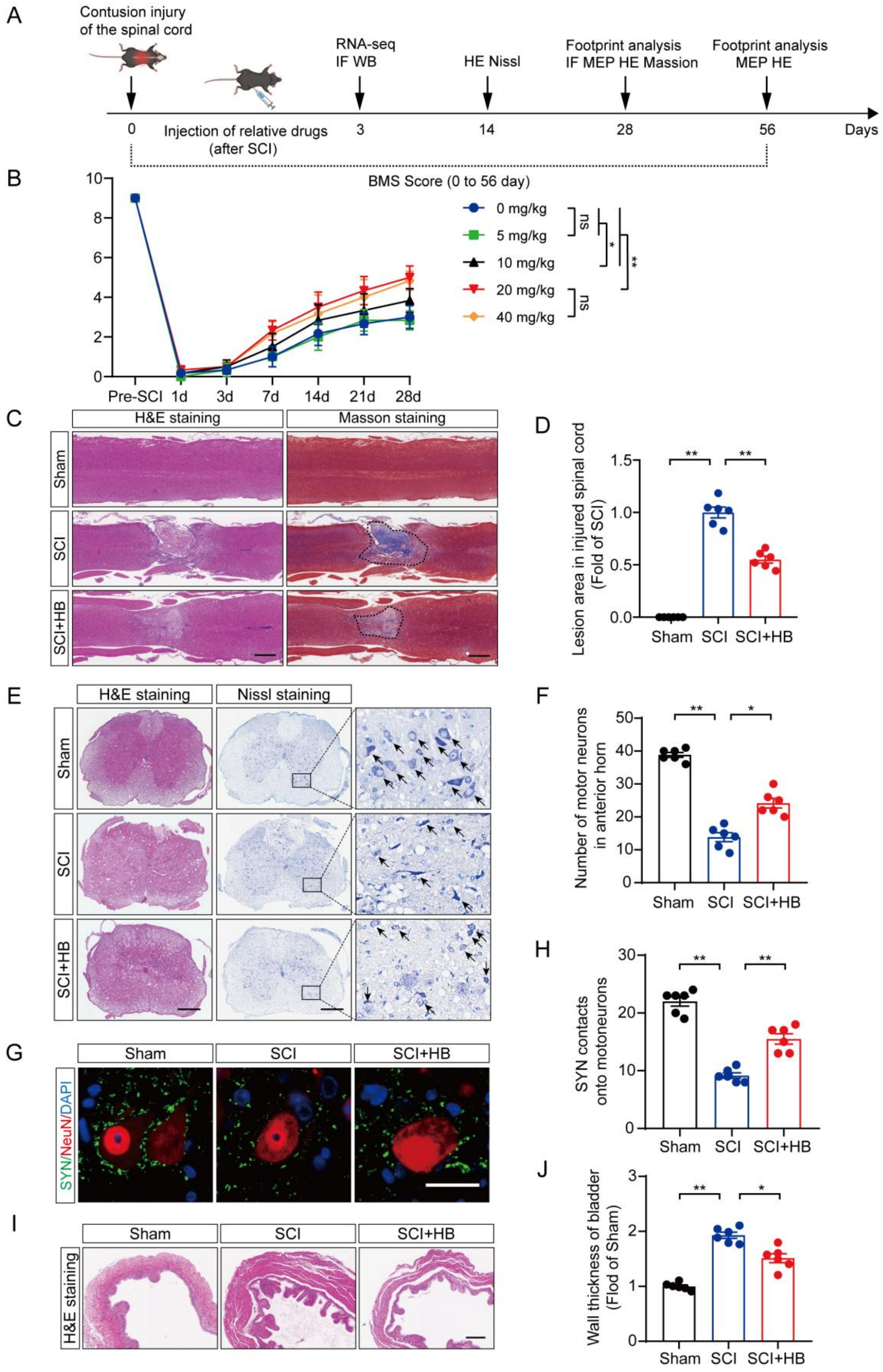

Figure S1. HB promotes functional recovery after SCI. (A) The Flow chart of animal experiments. **(B)** Dose–response chart showing the optimal dosage of HB (20 mg/kg) for 28 days after SCI for evaluation of BMS score. **(C)** Spinal cords in each

group were taken for longitudinal section on day 28 after SCI and examined by HE and Masson staining (scale bar: 500 μm). **(D)** Evaluation of Masson-stained lesion within the spinal cords across all groups. **(E)** Transverse spinal cord sections from each group were analyzed by HE staining and Nissl staining on day 14 after SCI; black arrows indicating the motoneurons; scale bar: 500 μm. **(F)** Quantitative analysis of Nissl positive motor neurons in the anterior horn of spinal cord from each group. **(G)** Representative images of spinal cord sections below the injury (T10-T12) and stained on day 28 after SCI with antibodies against SYN/NeuN; scale bar: 5 μm. **(H)** Relevant quantification of the number of synapses contacting onto motor neurons. **(I)** The H&E staining of the bladder from each group. scale bar: 500 μm. **(J)** The wall thickness of the bladder in each group, showing that the bladder function recovered faster in the HB treatment group. The data are presented as the means ± SEM (n = 6 mice per group); ns stands for not significant, *$P < 0.05$, **$P < 0.01$.

Figure S2

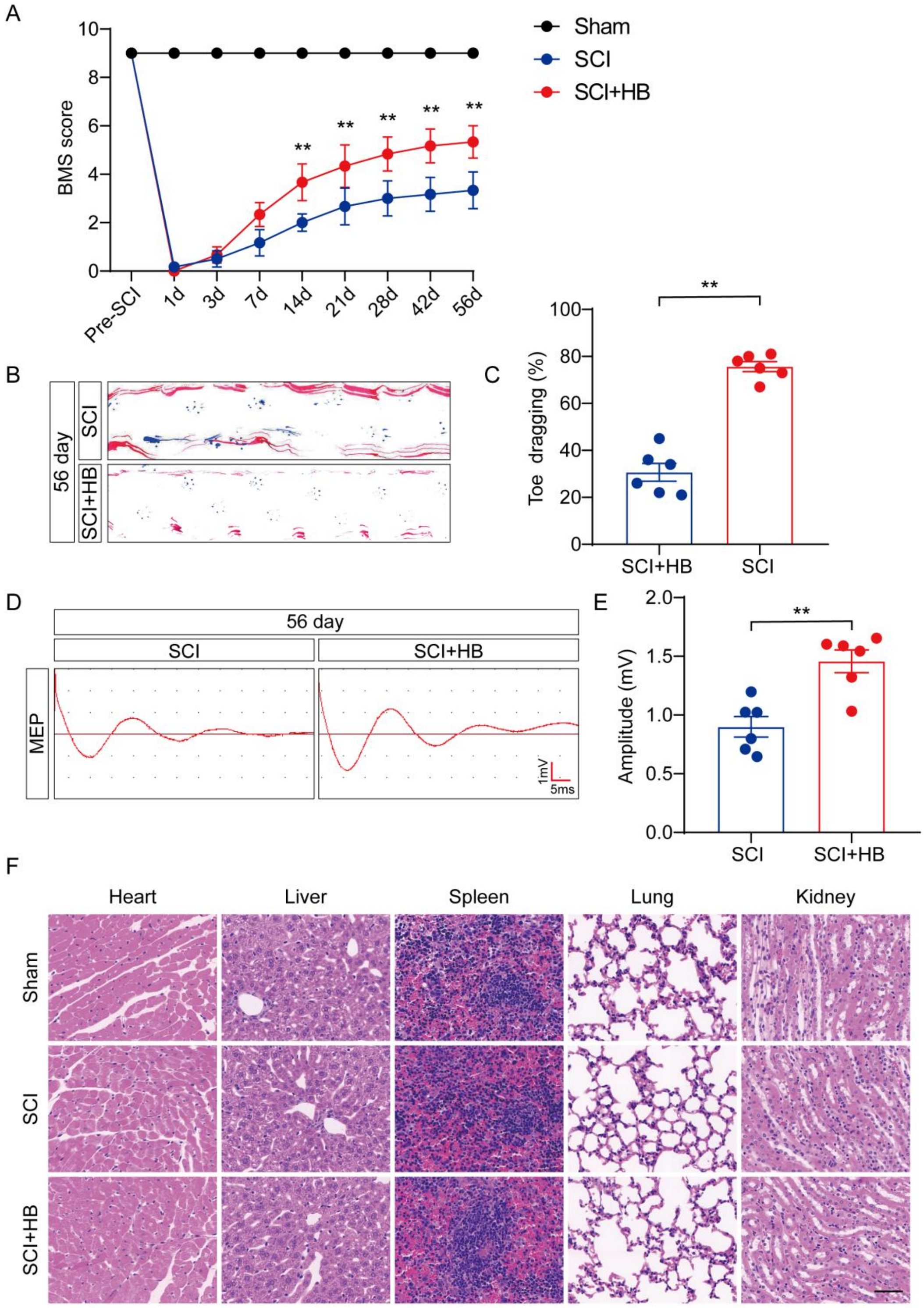

Figure S2. Long-term effects of HB on functional recovery and safety assessment. **(A)** BMS score of Sham, SCI, and SCI + HB group on days 1, 3, 7, 14, 21, 28, 42 and 56 after SCI. **(B)** Representative images of footprints used in walking analyses of

mice on day 56 after SCI. Blue: forepaw print; Red: hindpaw print. **(C)** Toe dragging (%) analyses of mice on day 56 in the respective groups. **(D)** Representative diagrams of motor evoked potential (MEP) detection in 56 days after SCI from SCI, and SCI + HB group. **(E)** Quantitative analysis of the amplitude of the first peak (mV). **(F)** HE staining of heart, liver, spleen, lung, and kidney tissues from Sham, SCI, and SCI + HB group. Scale bar: 50 μm. Independent-sample t-tests were employed to determine remarkably significant differences between two groups. The data are presented as the means ± SEM (n = 6 mice per group); *$P < 0.05$, **$P < 0.01$.

Figure S3

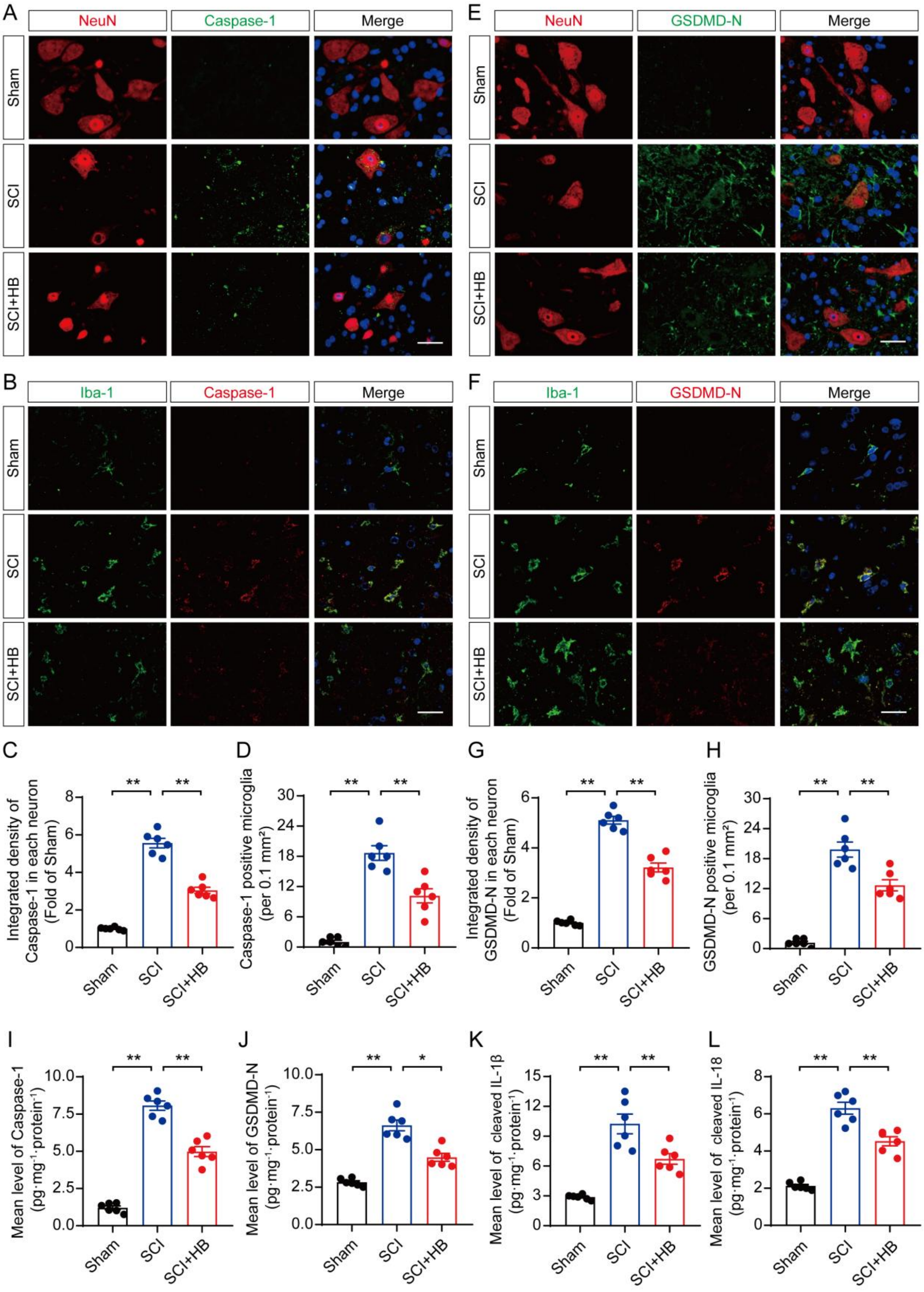

Figure S3. HB attenuates pyroptosis after SCI. (A) Typical immunofluorescence staining images of the spinal cord ventral horn grey matter showing caspase-1 and NeuN colocalization in the indicated groups on day 3 after SCI; scale bar: 20 μm. **(B)**

Typical immunofluorescence staining images of the spinal cord ventral horn grey matter showing caspase-1 and Iba-1 colocalization in the indicated groups on day 3 after SCI; scale bar: 20 μm. **(C)** Graph showing the relative intensities of caspase-1 immunofluorescence staining in neurons from the indicated groups. **(D)** Graph showing the number of caspase-1-positive microglia in the indicated groups. **(E)** Typical immunofluorescence staining images of the spinal cord ventral horn grey matter showing GSDMD-N and NeuN colocalization in the indicated groups on day 3 after SCI; scale bar: 20 μm. **(F)** Typical immunofluorescence staining images of the spinal cord ventral horn grey matter showing GSDMD-N and Iba-1 colocalization in the indicated groups on day 3 after SCI; scale bar: 20 μm. **(G)** Graph showing the relative intensities of GSDMD-N immunofluorescence staining in neurons from the indicated groups. **(H)** Graph showing the number of GSDMD-N-positive microglia in the indicated groups. **(I-L)** The quantities of Caspase-1, GSDMD-N, cleaved IL-1β and cleaved IL-18 in the injured spinal cord segments were detected by ELISA. The data are presented as the means ± SEM (n = 6 mice per group); $*P < 0.05$, $**P < 0.01$.

Figure S4

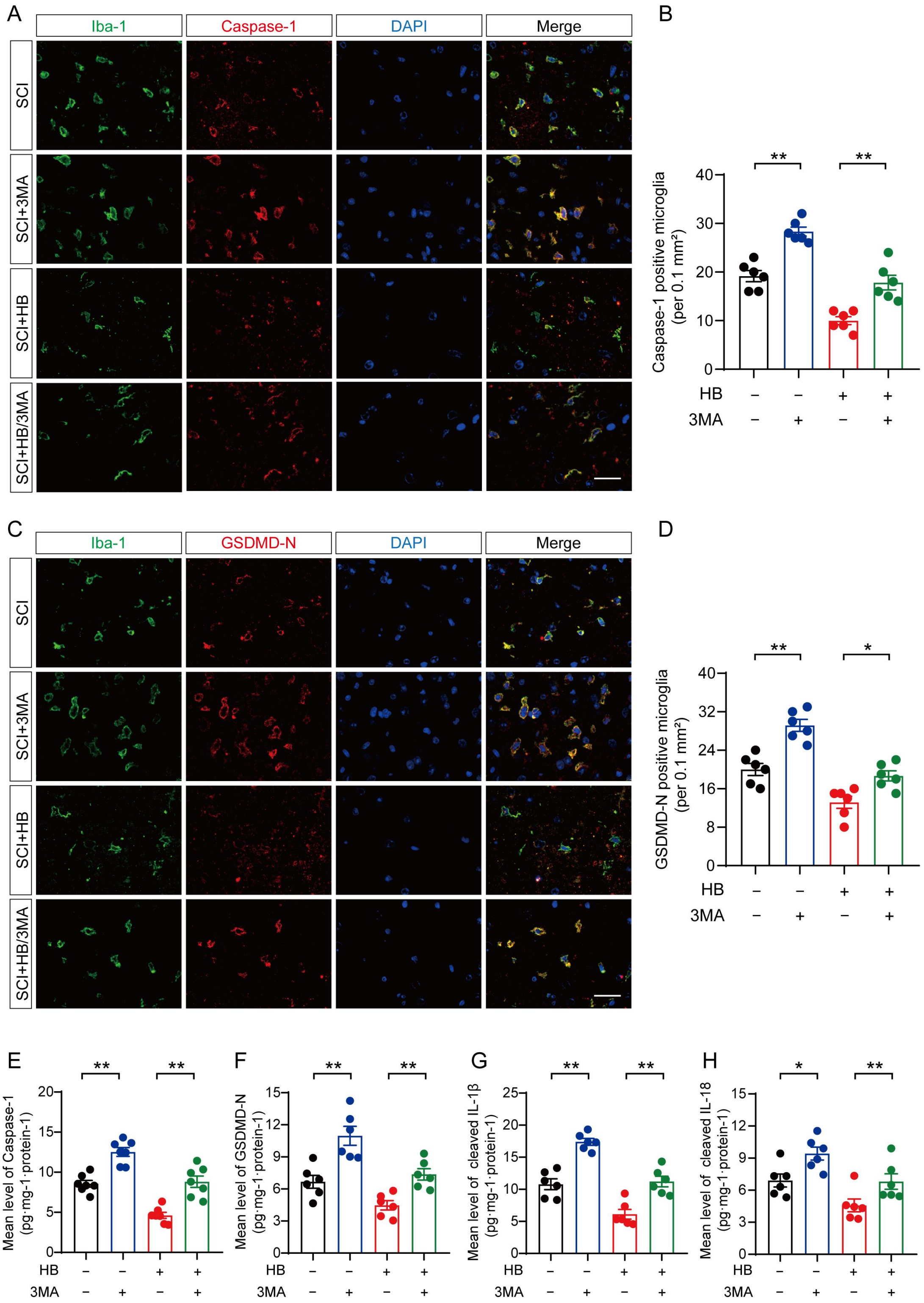

Figure S4. HB inhibits pyroptosis by activating autophagy after SCI. (A) Typical immunofluorescence staining images of the spinal cord ventral horn grey matter showing caspase-1 and Iba-1 colocalization in the indicated groups on day 3 after SCI;

scale bar: 20 μm. **(B)** Graph showing the number of caspase-1-positive microglia in the indicated groups. **(C)** Typical immunofluorescence staining images of the spinal cord ventral horn grey matter showing GSDMD-N and Iba-1 colocalization in the indicated groups on day 3 after SCI; scale bar: 20 μm. **(D)** Graph showing the number of GSDMD-N-positive microglia in the indicated groups. **(E-H)** The quantities of Caspase-1, GSDMD-N, cleaved IL-1β and cleaved IL-18 in the injured spinal cord segments were detected by ELISA. The data are presented as the means ± SEM (n = 6 mice per group); *P < 0.05, **P < 0.01.

Figure S5

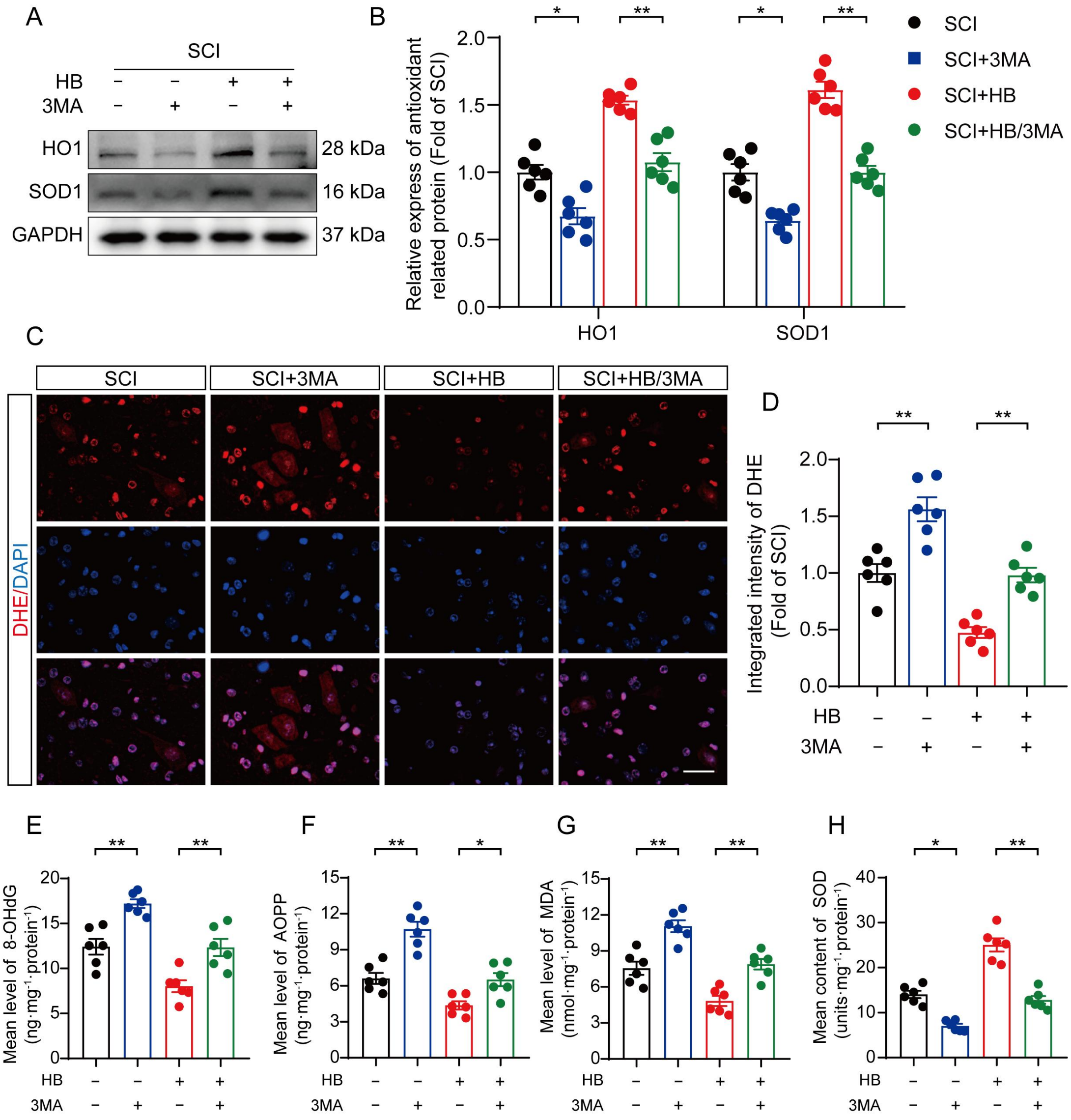

Figure S5. HB inhibits oxidative stress by activating autophagy after SCI. (A-B) WB analysis and quantification of HO1 and SOD1 protein levels in spinal cord lesions from the indicated groups on day 3 after SCI. GAPDH was utilized as a loading control. **(C)** Frozen sections of spinal cord from each group were stained with DHE (red; a ROS fluorescent probe). DAPI staining for nuclei (blue; scale bar: 20 μm). **(D)** A quantification graph for DHE immunofluorescence is shown in right. **(E-G)** The quantities of 8-OHdG, AOPP and MDA in the injured spinal cord segments were detected by ELISA. **(H)** The enzyme content of SOD in the injured spinal cord segments were detected by ELISA. The data are presented as the means ±

SEM (n = 6 mice per group); *P < 0.05, **P < 0.01.

Figure S6

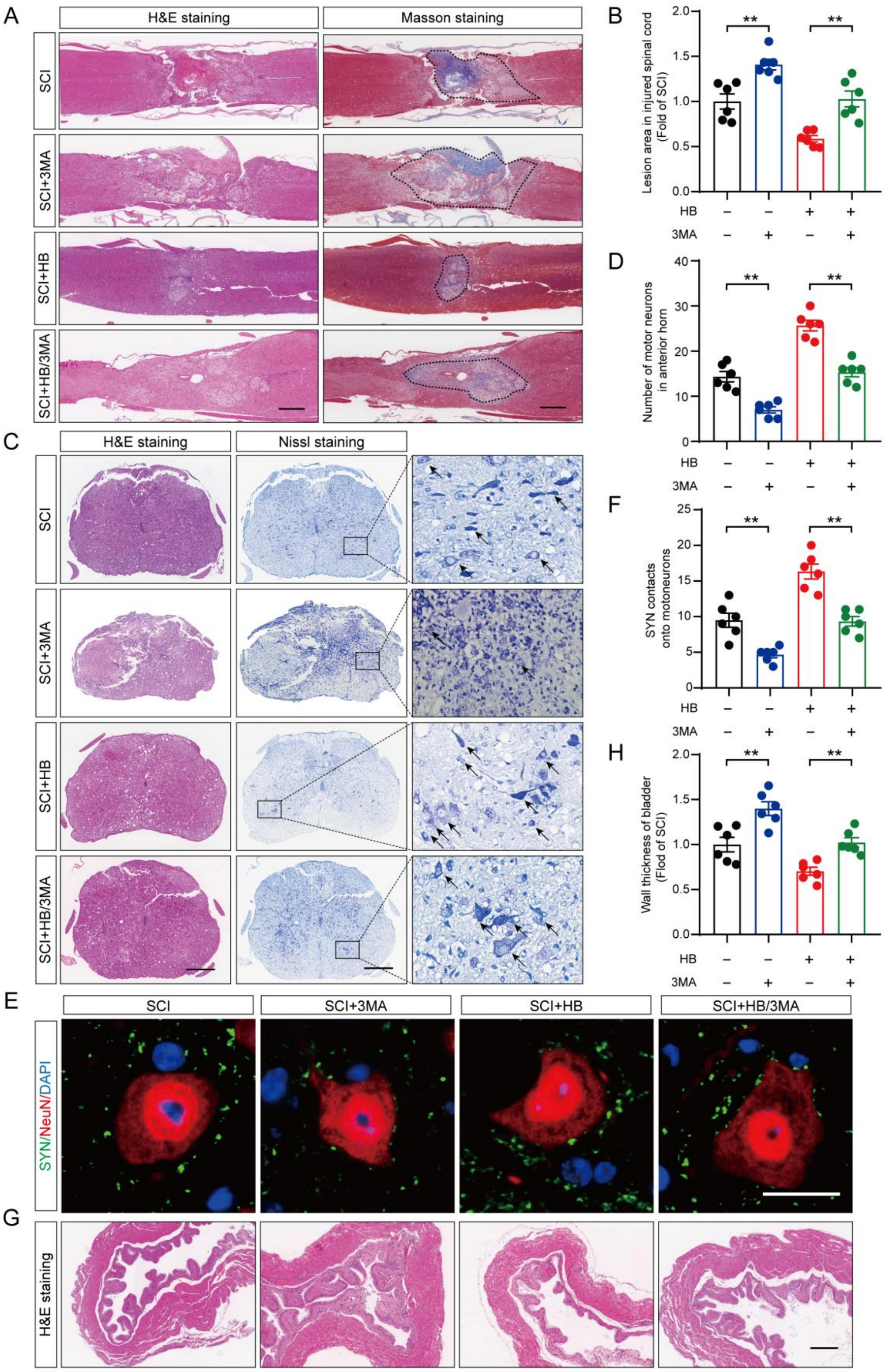

Figure S6. HB enhances functional recovery by promoting autophagy. (A) Spinal cords in each group were taken for longitudinal section on day 28 after SCI and examined by HE and Masson staining (scale bar: 500 μm). **(B)** Evaluation of

Masson-stained lesion within the spinal cords across all groups. **(C)** Transverse spinal cord sections from each group were analyzed by HE staining and Nissl staining on day 14 after SCI; black arrows indicating the motoneurons; scale bar: 500 μm. **(D)** Quantitative analysis of Nissl positive motor neurons in the anterior horn of spinal cord from each group. **(E)** Representative images of spinal cord sections below the injury (T10-T12) and stained on day 28 after SCI with antibodies against SYN/NeuN; scale bar: 5 μm. **(F)** Relevant quantification of the number of synapses contacting onto motor neurons. **(G)** The H&E staining of the bladder from each group. scale bar: 500 μm. **(H)** The wall thickness of the bladder in each group, showing that the bladder function recovered faster in the HB treatment group. The data are presented as the means ± SEM (n = 6 mice per group); *$P < 0.05$, **$P < 0.01$.

Figure S7

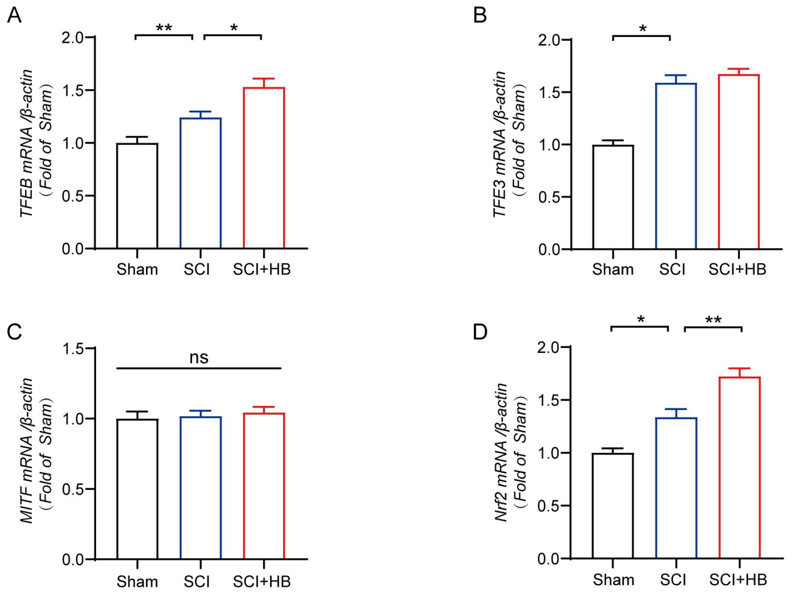

Figure S7. Expression of MiTF/TFE family transcription factors and Nrf2 in spinal cord treated with HB. (A-C) Relative mRNA level of *Tfeb*, *Tfe3* and *Mitf* in the spinal cord lesions from Sham, SCI, and SCI + HB group normalized to control β-actin. **(D)** Relative mRNA level of *Nrf2* in the spinal cord lesions from Sham, SCI, and SCI + HB group normalized to control β-actin. The data are presented as the means ± SEM (n = 6 mice per group); ns stands for not significant, *P < 0.05, **P < 0.01.

Figure S8

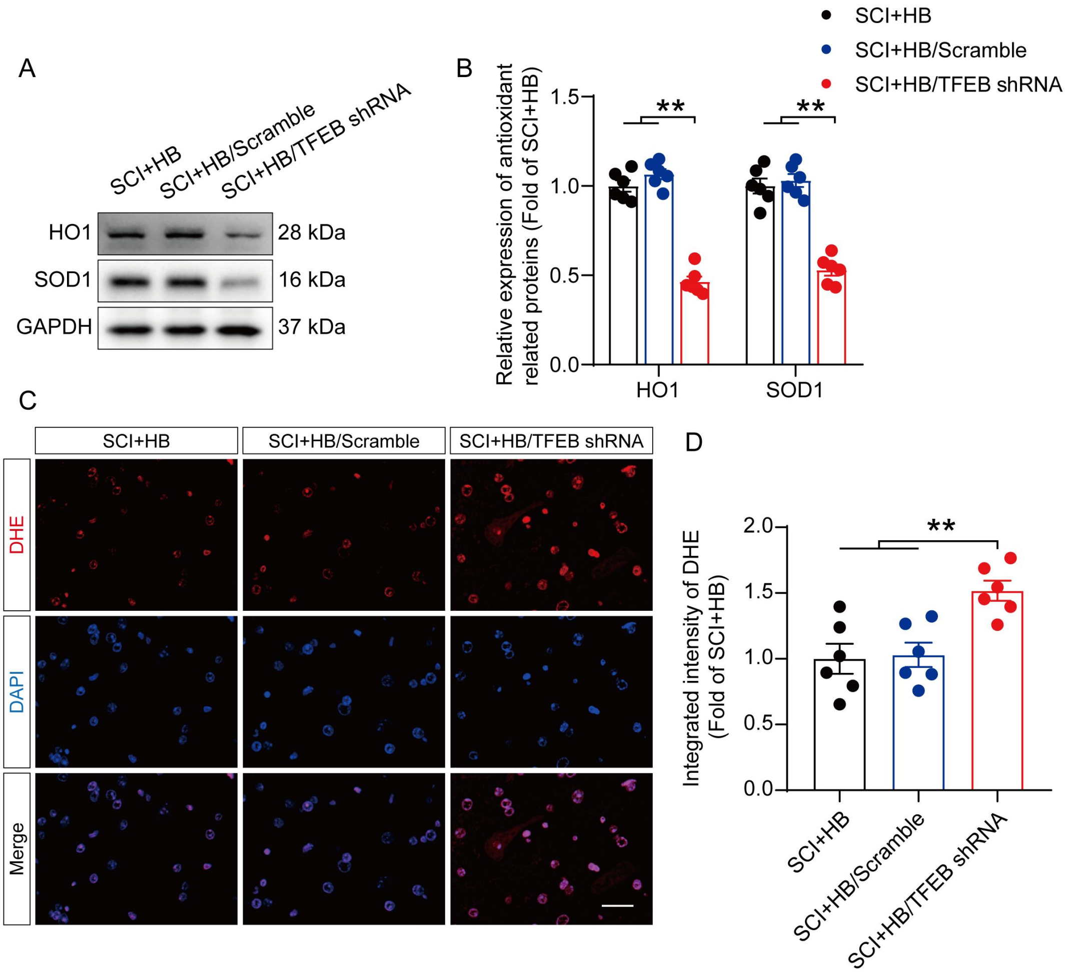

Figure S8. HB suppresses oxidative stress by upregulating TFEB activity after SCI. (A-B) WB analysis and quantification of HO1 and SOD1 protein levels in spinal cord lesions from the indicated groups on day 3 after SCI. GAPDH was utilized as a loading control. **(C)** Frozen sections of spinal cord from each group were stained with DHE (red; a ROS fluorescent probe). DAPI staining for nuclei (blue; scale bar: 20 μm). **(D)** Quantified integrated intensity of DHE in each group. The data are presented as the means ± SEM (n = 6 mice per group); **P* < 0.05, ***P* < 0.01.

Figure S9

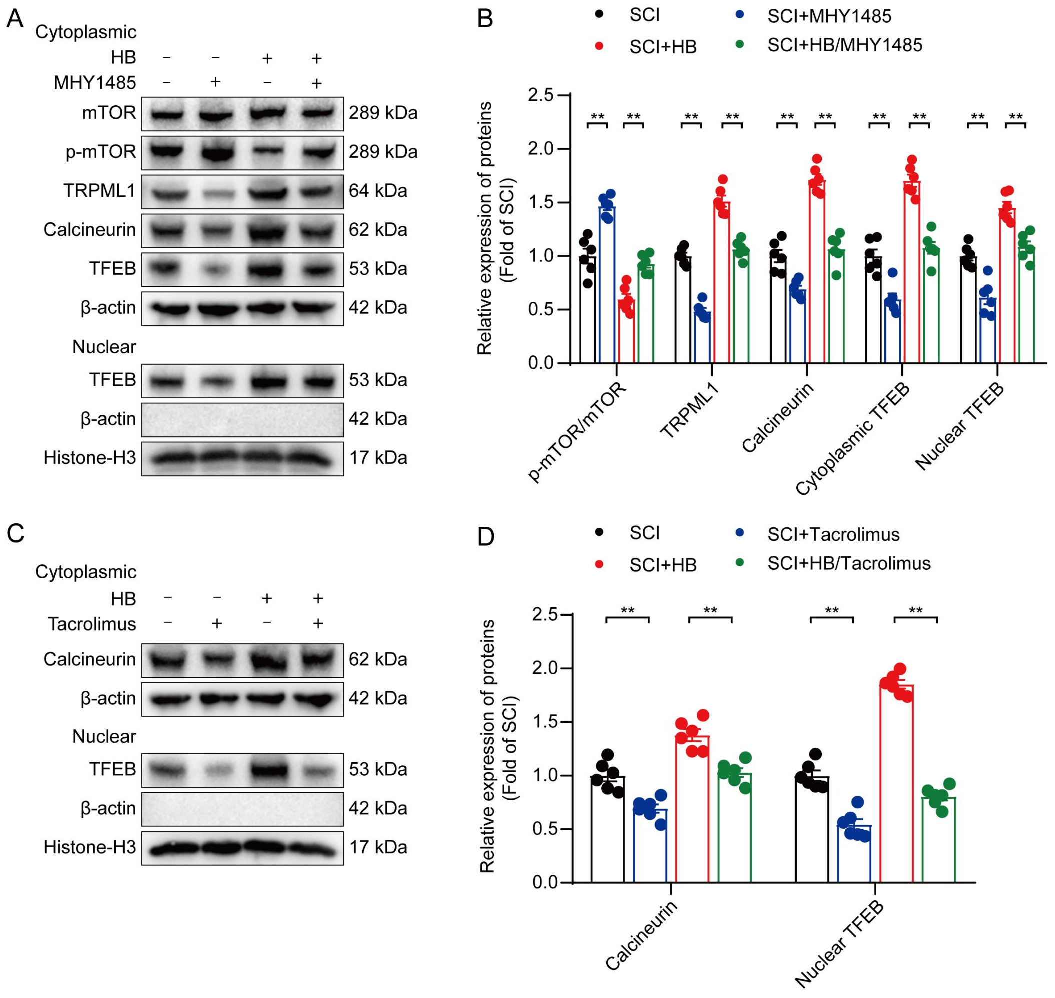

Figure S9. HB activates TFEB through the AMPK-TRPML1-calcineurin signaling pathway. (A-B) WB analysis of mTOR, p-mTOR, TRPML1, calcineurin, cytoplasmic and nuclear TFEB in the SCI, SCI + MHY1485, SCI + HB and SCI + HB/MHY1485 groups. β-actin and Histone-H3 was used as a loading control. The densitometry quantification shown on the right reveals that MHY1485 inhibited the HB-induced effects. **(C-D)** Representative western blot analysis of Calcineurin and TFEB in the injured spinal cord lesion from SCI, SCI + Tacrolimus, SCI + HB, and SCI + HB/Tacrolimus groups. β-actin or Histone-H3 was used as controls. The graphs on the right show the summary data from western blots. The data are presented as the means ± SEM (n = 6 mice per group); *$P < 0.05$, **$P < 0.01$.

Tables

Primer name	Primer sequences
Tfeb	*5'- CAGCAGGTGGTGAAGCAAGAGT-3' (forward)*
	5'-TCCAGGTGATGGAACGGAGACT-3'(reverse)
Tfe3	*5'-CCAGGCTCAGGAACAGGAGA-3' (forward)*
	5'-TACTGTTTGACCTGCTGCCG-3' (reverse)
Mitf	*5'-CATTCTCAAGGCCTCTGTGGACTA-3' (forward)*
	5'-GTGCCGAGGTTGTTGGTAAAGGTG-3' (reverse)
Nrf2	*5'-TTCCTCTGCTGCCATTAGTCAGTC-3' (forward)*
	5'-GCTCTTCCATTTCCGAGTCACTG' (reverse)
β-actin	*5'-GGCTCCTAGCACCATGAAGA-3' (forward)*
	5'-AGCTCAGTAACAGTCCGCC-3' (reverse)

Table S1. Information of the primer sequences for qPCR.
